# Modeling and Evaluating Integrated Pollution Control Measures in Rivers: A Case Study of the Lianjiang River Basin

**DOI:** 10.3390/toxics14030216

**Published:** 2026-03-01

**Authors:** Jinxi Zheng, Yongyou Hu, Wenqin Xu, Shewei Yang, Xiangzhuan Zeng, Youshun Guo, Zhenjiang Yu, Jianhua Cheng

**Affiliations:** 1 Key Laboratory of Pollution Control and Ecological Restoration in Industrial Clusters, Ministry of Education, College of Environment and Energy, South China University of Technology, Guangzhou 510006, China; 2 Guangdong Yifang Environmental Protection Technology Co., Ltd., Guangzhou 510623, China; 3 Guangdong Provincial Academy of Environmental Science, Guangzhou 510045, China; 4 Guangdong Enweile Environmental Technological Co., Ltd., Foshan 528000, China

**Keywords:** water quality pollution, watershed management, MIKE 21 model, multi-source interception, pollutant reduction

## Abstract

With rapid industrialization and urbanization, water pollution in urban rivers has become increasingly severe, posing significant threats to regional ecological environments and water security. The Puning section of the Lianjiang River in Guangdong Province, China, suffers from complex pollution originating from multiple sources, including domestic sewage, industrial wastewater, and agricultural non-point source pollution. This underscores an urgent need for integrated river pollution control in the region. In this study, a coupled hydrodynamic-water quality model was established to systematically analyze and simulate water quality conditions in the Puning section. A total of 22 pollution control scenarios were proposed and evaluated. The results indicated that most monitoring sections in the Puning segment failed to meet the Class V surface water quality standards, with notably high concentrations of chemical oxygen demand (COD), ammonia nitrogen (NH_3_-N), and total phosphorus (TP). Numerical simulations revealed that the multi-source interception scenario—simultaneously intercepting inflows at Tiande Bridge (Baikeng Lake), Dayangmei (Liusha Zhonghe), and Liudoupu (Liusha Zhonghe)—performed best in reducing pollutant concentrations. Specifically, this scenario achieved reductions exceeding 90% in NH_3_-N and TP concentrations. Furthermore, the study demonstrates that for basins with complex pollution sources, integrated management strategies—including the construction of wastewater treatment facilities, control of point and non-point sources, and ecological restoration measures—can effectively improve water quality and optimize the water environmental capacity. These findings provide theoretical and technical support for water environment management in the Lianjiang River Basin and offer valuable insights for water quality management in similar regions.

## 1. Introduction

Rivers are vital components of natural ecosystems, playing an irreplaceable role in ensuring regional water supply, maintaining ecological balance, and promoting socio-economic development [1]. However, intensified human activities and climate change have led to the discharge of substantial quantities of domestic sewage, industrial wastewater, and agricultural non-point source pollutants into rivers. This has resulted in water quality degradation and triggering numerous adverse events, which have garnered widespread concern [2]. The United Nations 2030 Sustainable Development Goals emphasize the importance of protecting water bodies and maintaining water quality [3,4]. Consequently, due to ongoing population growth and rapid urbanization, close and continuous assessment of river water quality is imperative.

To effectively reduce the concentrations of pollutants such as chemical oxygen demand (COD), biochemical oxygen demand (BOD), ammonia nitrogen (NH_3_-N), and total phosphorus (TP) in water bodies, treatment prior to discharge into natural waters is typically required, a process accomplished by wastewater treatment plants [5]. However, even when these facilities meet discharge standards, the complexity of the wastewater often results in residual pollutants, which may pose ecological risks and disrupt aquatic ecosystems [6,7,8]. Furthermore, with continuing urbanization and agricultural activities, pollution sources in river basins have become increasingly diverse and complex [9]. In addition to point source pollution from urban domestic sewage and industrial effluents, dispersed discharges from agricultural production, rural households, and land use changes are on the rise, creating a multi-source, interactive pollution pattern within watersheds [10]. In this context, individual wastewater treatment measures are insufficient to address the complex pollution challenges; comprehensive integrated management strategies are required, encompassing point including point source pollution control, non-point source pollution management, ecological water replenishment, and upgrades to wastewater treatment facilities. The lack of hydrological data and inadequate analysis of pollution mechanisms often leads to relatively rudimentary management measures for small rivers, making treatment difficult and rendering water bodies prone to blackening, odor, and other issues [11]. Therefore, developing appropriate water environment simulation and analysis systems and proposing optimal management strategies for these medium and small-sized watersheds holds significant practical and economic importance.

In recent years, the simulation and analysis of river pollution using numerical models has gained considerable attention [12]. River water quality models such as QUAL2K [13,14,15], MIKE 21 [16], HEC-RAS [17], and TOMCAT [18] effectively simulate and analyze the evolution of pollutant behavior in water bodies by considering flow rates and pollutant loads to analyze hydrodynamic pathways and water quality processes within river networks, either individually or simultaneously [19]. QUAL2K offers advantages in simulating pollutant transport and transformation in data-sparse regions, but its limitation lies in its primary applicability to one-dimensional steady-state simulations in rivers and streams, making it difficult to handle two-dimensional lateral dispersion in complex terrains. HEC-RAS performs excellently in flood inundation simulation and water surface profile calculations; however, its versatility as a water quality model is relatively limited, its code is not open-source, technical support for non-US users is lacking, and its water quality simulation capabilities are not widely adopted. Models like TOMCAT have been applied in specific studies, but their overall applicability varies depending on watershed characteristics. The MIKE 21 model, with its strong coupling capabilities and visualization functions, is widely applied in simulating hydrodynamics, water quality, flood, sediment transport, and ecosystems. It is suitable for simulating and analyzing complex water bodies such as rivers, lakes, estuaries, and coastal areas, providing an effective tool for scenario simulation in water pollution control [20]. Current numerical simulation studies focusing on conventional pollutants in small rivers often concentrate on the transport pathways and reduction mechanisms of pollutants within specific areas [21,22], with limited research analyzing changes in pollutant transport under multiple scenarios encompassing source control and process reduction. Such analyses could provide crucial guidance for the siting and construction of pollution control facilities within watersheds.

Lianjiang is an important watercourse in eastern Guangdong region, flowing through areas such as Jieyang and Puning, serving as a vital water source for economic activities, agricultural irrigation, and residential life. The Lianjiang River basin is densely populated with frequent socio-economic activities, and its water environment plays a crucial role in supporting regional development [23]. However, due to factors such as a complex industrial structure, insufficient wastewater treatment capacity, and a lack of basin-wide management, water pollution in the Lianjiang River basin has become a prominent issue [11]. Therefore, conducting a comprehensive investigation into the current water environment, pollution source diagnostics, and integrated pollution control technologies for the Puning section of the Lianjiang River is not only significant for improving local water quality and ensuring public safety but also has profound implications for promoting regional green development and fostering ecological civilization construction within the watershed. This study focuses on the Puning section of the Lianjiang River basin. Initially, the water quality pollution status of the main river and its tributaries in the Puning section was assessed. Subsequently, based on the MIKE 21 hydrodynamic and water quality model, three distinct water quality pollution control scenarios were proposed, leading to the design of 22 management strategies. Numerical simulations were conducted to analyze the distribution of COD, NH_3_-N, and TP concentrations, and to evaluate the effectiveness of each scenario in reducing pollutant concentrations. This study assesses the comprehensive effects of multi-source pollution control strategies in the Lianjiang River Basin, exploring the superiority of integrated measures (including interception, engineering interventions, and ecological restoration) over single measures—that is, an integrated approach.

## 2. Data and Methods

### 2.1. Overview of the Study Area

**Figure 1:**
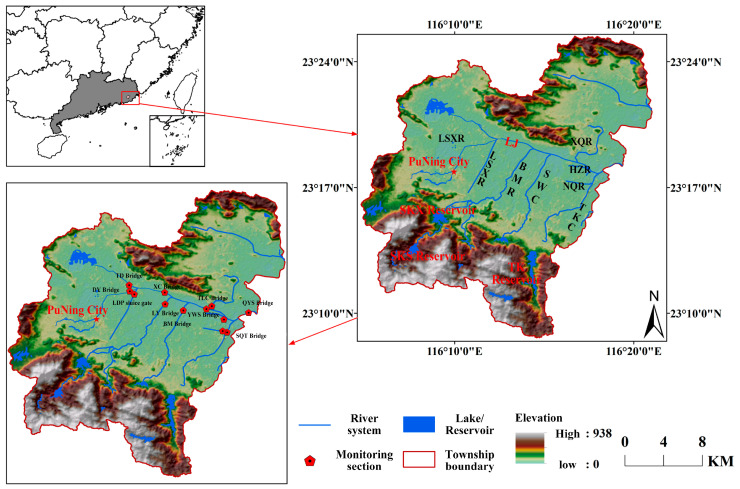
Geographical distribution map of the Puning section of the Lianjiang River basin and monitoring sections.

The Lianjiang River basin, located in the eastern part of Guangdong Province, is one of the three major river basins in the Chaoshan region. Its main river originates from Puning in the northwest of Jieyang, flows southeast through Chaonan and Chaoyang districts of Shantou, and collects various tributaries such as Qiufeng River and Beigang River along the way before emptying into the South China Sea at Haimen Town (Figure 1). The basin is shaped like a palm fan, surrounded by high mountains and hills, with fertile alluvial plains scattered throughout. The area of the mountainous and plain regions is almost equal. The middle reaches of the river feature the Lianjiang sluice gate in Tongyu, while the lower reaches at the Chaoyang district have the Haimen Bay Bridge sluice gate at the river mouth. Situated south of the Tropic of Cancer, the basin spans longitudes 116°5′31″ to 116°36′21″ E and latitudes 23°6′45″ to 23°23′34″ N, with an average width of 26 km from north to south and an average length of 52 km from east to west. The total basin area is 1353.5 km^2^ [23], with 838.5 km^2^ in Shantou City and 515 km^2^ in Puning City. The average annual rainfall in the basin is 1872 mm, with an average annual water yield of 1 million m^3^/km^2^. The instantaneous flow rate varies between 0 and 1324 m^3^/s, with a multi-year average of 1.35 billion m^3^, showing significant annual fluctuations in flow. The main river of the Lianjiang River in Puning City stretches for 29.8 km, with a drainage area of 515 km^2^. Nine primary tributaries join the Lianjiang River within Puning, including Liushaxin River (LSXR), Liushazhong River (LSZR), Bai Keng Lake River (BKLR), Qilin Creek (QLC), Nanyang Creek (NYC), Bai Ma River, (BMR), Shuiwei Creek (SWC), Tangkeng Creek (TKC), and Xiqie River (XQR). The river passes through 15 towns and streets, with a permanent population of 1.75 million. The main pollution sources are domestic and agricultural.

### 2.2. Data Sources and Sample Collection

The multi-source data used in this study mainly include: Digital Elevation Model (DEM), land use, meteorological data (primarily annual average precipitation and temperature), population density, night-time lights, number of industrial enterprises, livestock and poultry farming data for Puning City, fertilizer application data for Puning City, year-end permanent population and national economic data, and water quality monitoring data for the main river and tributaries of the Lianjiang River in Puning from 2016 to 2018. Specifically, the DEM data were obtained from the Geospatial Cloud Data Platform (https://www.gscloud.cn/). Precipitation and temperature raster data were sourced from the National Qinghai–Tibet Plateau Data Center [24]. The annual land use data from 1990 to 2020 were derived from the dataset constructed by [25], which classifies Chinese land use into nine categories: farmland, forest land, shrubland, grassland, water bodies, snow/ice, barren land, impervious surfaces, and wetlands. Based on the characteristics of the study area and research objectives, this study reclassified the original land use types (combining shrubland and forest land as forest land, merging water bodies and wetlands as water bodies, and combining snow/ice and barren land as unused land) in ArcGIS 10.8. The final classification resulted in six land use types: forest land, grassland, cultivated land, urban land, water bodies, and unused land. Population density data were sourced from the 1990–2022 global population density dataset developed by [26]. Night-time lights data were obtained from the National Qinghai–Tibet Plateau Science Data Center [27]. The number of industrial enterprises, livestock and poultry farming data, fertilizer application data, year-end permanent population, and national economic data for Puning City were sourced from the Puning City Statistical Yearbook (http://www.puning.gov.cn/, accessed on 25 Feruary 2026).

In accordance with the “Water Quality—Sampling Techniques Guidelines” (HJ 494-2009) [28], water samples were collected from 12 monitoring sections of the main and tributary rivers in the Puning section of the Lianjiang River basin between January 2016 and December 2018 (Figure 1). The samples were collected using an acrylic water sampler placed 0.3 m below the water surface. The samples were collected in polyethylene bottles, pre-washed three times (100 mL per sample, with three samples taken at each site), and stored in a refrigerator (0–4 °C). The monitoring parameters included dissolved oxygen (DO), NH_3_-N, TP, and COD. DO was measured on-site, while the other parameters were tested in the laboratory within 48 h of sampling. DO was determined using a portable DO analyzer (HQ30D, HACH, Loveland, CO, USA). NH_3_-N was measured using the Nessler reagent spectrophotometric method (HJ 535-2009) [29], TP was quantified using the ammonium molybdate spectrophotometric method (GB/T 11893-89) [30], and COD was measured using the rapid digestion spectrophotometric method (HJ/T 399-2007) [31].

In this study, water quality assessment and target setting were conducted in accordance with the Class V standards of the Chinese Environmental Quality Standards for Surface Water (GB 3838-2002) [32]. The corresponding threshold values are as follows: COD ≤ 40 mg/L, NH_3_-N ≤ 2.0 mg/L, TP ≤ 0.4 mg/L, and DO ≥ 2 mg/L.

### 2.3. MIKE 21 Model Setup

#### 2.3.1. MIKE 21 Hydrodynamic Module

MIKE 21 is a well-established two-dimensional, coupled hydrodynamic–water quality modeling system developed by the Danish Hydraulic Institute (DHI). It has been widely applied to rivers, lakes, estuaries, and coastal embayments, and is capable of simulating hydrodynamic processes (e.g., tides, currents, and storm surges) as well as key aquatic environmental components (e.g., heat transport, salinity dynamics, water quality variations, wave-induced disturbances, and sediment transport and deposition) with high accuracy. Owing to these capabilities, MIKE 21 has been extensively used in river hydraulics and hydrology studies, water environment management, and water resources planning and management [21].

Given the relatively large channel width in the urban Puning section of the Lianjiang River (exceeding 150 m), pollutant transport exhibits pronounced lateral dispersion and cannot be assumed to be fully mixed over short distances. Therefore, the MIKE 21 two-dimensional coupled hydrodynamic–water quality model, implemented on the MIKE Zero platform, was selected to simulate hydrodynamic conditions and associated water quality dynamics in the Lianjiang River basin.

The hydrodynamic (HD) module in MIKE 21 is formulated from the three-dimensional incompressible Reynolds-averaged Navier–Stokes equations, with the Boussinesq approximation and the hydrostatic pressure assumption. The HD module consists of the continuity equation and the momentum equations, and the governing equation set is expressed as follows:

(1) $$\frac{\partial h}{\partial t}+\frac{\partial hu}{\partial x}+\frac{\partial hv}{\partial y}=hS$$

(2) $$\begin{array}{c} \frac{\partial hu}{\partial t}+\frac{\partial hu^{2}}{\partial x}+\frac{\partial hu\nu }{\partial y}=f\nu h-gh\frac{\partial \eta }{\partial x}-\frac{h}{\rho_{0}}\frac{\partial p_{a}}{\partial x}-\frac{gh^{2}}{2\rho_{0}}\frac{\partial \rho }{\partial x}\\ +\frac{\tau_{sx}}{\rho_{0}}-\frac{\tau_{bx}}{\rho_{0}}-\frac{1}{\rho_{0}}\left(\frac{\partial S_{xx}}{\partial x}+\frac{\partial S_{xy}}{\partial y}\right)+\frac{\partial }{\partial x}\left(hT_{xx}\right)+\frac{\partial }{\partial y}\left(hT_{xy}\right)+hu_{s}S \end{array}$$

(3) $$\begin{array}{c} \frac{\partial h\nu }{\partial t}+\frac{\partial hu\nu }{\partial x}+\frac{\partial h\nu^{2}}{\partial y}=-fuh-gh\frac{\partial \eta }{\partial y}-\frac{h}{\rho_{0}}\frac{\partial p_{a}}{\partial y}-\frac{gh^{2}}{2\rho_{0}}\frac{\partial \rho }{\partial y}\\ +\frac{\tau_{sy}}{\rho_{0}}-\frac{\tau_{by}}{\rho_{0}}-\frac{1}{\rho_{0}}\left(\frac{\partial S_{yx}}{\partial x}+\frac{\partial S_{yy}}{\partial y}\right)+\frac{\partial }{\partial x}\left(hT_{xy}\right)+\frac{\partial }{\partial y}\left(hT_{yy}\right)+h\nu_{s}S \end{array}$$

where t is time; η is the water surface elevation; d is the still-water depth; h=η+d is the total water depth; u and ν are the depth-averaged velocity components in the *x*- and *y*-directions; f is the Coriolis parameter; S denotes source/sink discharge; $S_{xx}$, $S_{xy}$, $S_{yx}$, and $S_{yy}$ are the components of the radiation stress tensor; $\tau_{sx}$ and $\tau_{sy}$ are the wind shear stresses at the free surface, whereas $\tau_{bx}$ and $\tau_{by}$ are the bed shear stresses; $T_{xx}$, $T_{xy}$, and $T_{yy}$ represent the lateral stress terms; and $u_{s}$ and $\nu_{s}$ are the velocity components associated with the source/sink terms.

#### 2.3.2. MIKE 21 Water Quality Module

The advection–dispersion (AD) module in MIKE 21 is implemented on the basis of the hydrodynamic (HD) module and employs the advection–dispersion equation to simulate the transport and spreading of constituents (e.g., salinity, heat, and pollutants) driven by advection and dispersion. The module can reproduce the spatiotemporal dynamics of conservative tracers (e.g., salinity) and, by specifying degradation/decay coefficients, can also represent non-conservative and degradable constituents (e.g., indicator bacteria and organic contaminants) in aquatic environments. Owing to its flexibility, the AD module has been widely applied in advanced water quality studies, including circulation-driven transport analyses, dispersion of conservative and non-conservative substances, and the simulation of pollutant spreading pathways [33].

The water quality module provides a mathematical framework for describing the transformation and fate of pollutants in aquatic environments and the interactions among controlling factors, and it represents a core component of water environment research. Therefore, the advection-dispersion (AD) module within MIKE 21 was employed to simulate pollutant transport and dispersion in the Lianjiang River basin. The governing equation is given as follows:

(4) $$\frac{\partial }{\partial t}\left(hC\right)+\frac{\partial }{\partial x}\left(uhC\right)+\frac{\partial }{\partial y}\left(\nu hC\right)=\frac{\partial }{\partial x}\left(hD_{x}\frac{\partial C}{\partial x}\right)+\frac{\partial }{\partial y}\left(hD_{y}\frac{\partial C}{\partial y}\right)+F\left(C\right)+S$$

where $D_{x}$ and $D_{y}$ are the turbulent dispersion coefficients in the *x*- and *y*-directions, respectively; C is the constituent concentration; and $F\left(C\right)$ represents the biochemical reaction term.

This research model adopts the quasi-steady state assumption and conducts simulations based on daily average flow and water quality data; the degradation coefficient is a lumped parameter, reflecting the combined effect of various natural purification processes. Details of terrain mesh generation, model parameterization, and model calibration and validation for MIKE 21 are provided in the Text S1 in Supplementary Material.

### 2.4. Water Environmental Capacity Estimation

Estimating water environmental capacity is an inherently integrative and interdisciplinary task, drawing on hydrology, chemistry, mathematics, and statistics. Its primary objective is to quantify the maximum pollutant load that a given water body (e.g., a river, lake, or reservoir) can assimilate without violating prescribed water quality standards. Reliable estimates of water environmental capacity underpin target-based water quality management and total pollutant load control, and are therefore critical for designing discharge control strategies and promoting the sustainable use of water resources. This assessment requires a comprehensive consideration of the system’s natural self-purification potential, hydrodynamic conditions, pollutant types and loads, and designated water uses, with the aim of aligning socio-economic development with the receiving water’s assimilative capacity and coordinating water resource development with environmental protection [34].

Based on the integrated monitoring dataset and the current-status assessment for the Puning section of the Lianjiang River basin, DO, COD, NH_3_-N, and TP were identified as the key control indicators. The water quality target for the Qingyangshan Bridge (QYSB) control unit was set to the Class V surface water standard, and the water environmental capacity was subsequently estimated. According to the designated water functional zoning and the configuration of monitoring sections, the study reach was delineated into one water environment control unit, with the QYSB section serving as the compliance section; the Xiachun Bridge (XCB), BMR, SWC, XQR, Nanqie River (NQR), Haizai River (HZR), TKC, and Yangweishan Bridge (YWSB) sections were used as routine monitoring sections to support baseline characterization and process-based analysis. Methodologically, considering data availability for hydrological inputs, channel morphology, and the complexity associated with multiple tributary confluences, an analytical approach was adopted for capacity calculations. Given the generally large width-to-depth ratio of the study reach (>20) and the potential for laterally non-uniform dispersion, a two-dimensional analytical model was selected (in accordance with the technical guideline for calculating assimilative capacity) to estimate the control-unit capacities for DO, COD, NH_3_-N, and TP. The corresponding equations are given as follows:

(5) $$W=\left[C_{s}e^{\frac{K_{x}}{86400u}}-C_{0}\right]\cdot h\cdot \sqrt{\pi \cdot M_{y}\cdot x\cdot u}\cdot \left[1+e^{\left(-\frac{u\cdot B^{2}}{M_{y}\cdot x}\right)}\right]\times 86.4$$

where W is the water environmental (assimilative) capacity (kg d^−1^); $C_{s}$ is the target concentration for the corresponding water quality parameter in the reach (mg/L); $C_{0}$ is the inflow concentration at the upstream boundary (mg/L); K denotes the integrated pollutant decay coefficient (d^−1^); u is the reach-averaged longitudinal velocity (m s^−1^); h is the mean water depth (m); *M_y_* is the transverse dispersion coefficient (m^2^ s^−1^); x is the control-unit length (km), and B is the channel width (m).

The remaining environmental capacity is defined as the difference between the total pollutant load that a river reach can assimilate and the pollutant load currently discharged into the reach. It is calculated as follows:

(6) $$W_{R}=W-W_{i}$$

where W is the water environmental (assimilative) capacity; $W_{i}$ is the total pollutant load currently discharged into the reach; and $W_{R}$ is the remaining water environmental capacity.

When $W_{R}$ > 0, the control unit still has assimilative capacity to receive additional pollutant loads while meeting the predefined water quality targets. In contrast, when $W_{R}$ < 0, the existing pollutant load exceeds the assimilative capacity of the control unit, indicating that load reductions are required to achieve water quality improvement.

**Figure 2:**
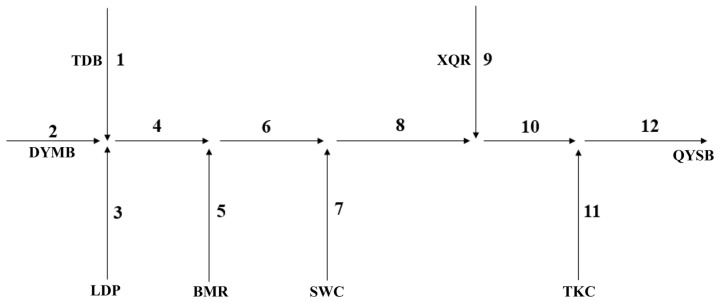
Schematic map of the control sections in the study area.

Based on the locations of outfalls, intake points, and tributary confluences, additional control sections were introduced to delineate the functional zone into 12 sub-reaches. Water environmental capacity was calculated for each sub-reach and then aggregated to obtain the total capacity for the functional zone. A schematic representation of the control sections in the study area is shown in Figure 2.

### 2.5. Statistical Analysis

The calibration and validation of the hydrodynamic module use the Nash–Sutcliffe Efficiency Coefficient (NSE) to evaluate the performance of the model. The calculation method is as follows:

(7) $$NSE=1-\left[\frac{\sum_{i=1}^{n}{\left(X_{i}^{obs}-X_{i}^{sim}\right)}^{2}}{\sum_{i=1}^{n}{\left(X_{i}^{obs}-X_{i}^{mean}\right)}^{2}}\right]$$

The water quality model uses Root Mean Square Error (RMSE) and Percent Bias (PBIAS) to evaluate the performance of the model. The calculation method is as follows:

(8) $$RMSE=\sqrt{\frac{1}{N}\sum_{i=1}^{n}{\left(X_{i}^{sim}-X_{i}^{obs}\right)}^{2}}$$

(9) $$PBIAS=\left[\frac{\sum_{i=1}^{n}\left(X_{i}^{obs}-X_{i}^{sim}\right)}{\sum_{i=1}^{n}\left(X_{i}^{obs}\right)}\times 100\right]$$

In the formula, n represents the number of monitoring indicators, $X_{i}^{obs}$ is the observed value of the i-th indicator, $X_{i}^{sim}$ is the simulated value of the i-th indicator, and $X_{i}^{mean}$ is the average of n observed values. It is generally believed that when the NSE is greater than 0.5, the PBIAS is less than or equal to 15%, and the RMSE is less than 10, the results of the model are reliable (Table 1).

## 3. Model Calibration and Validation

### 3.1. Hydrodynamic Model Calibration and Validation

**Figure 3:**
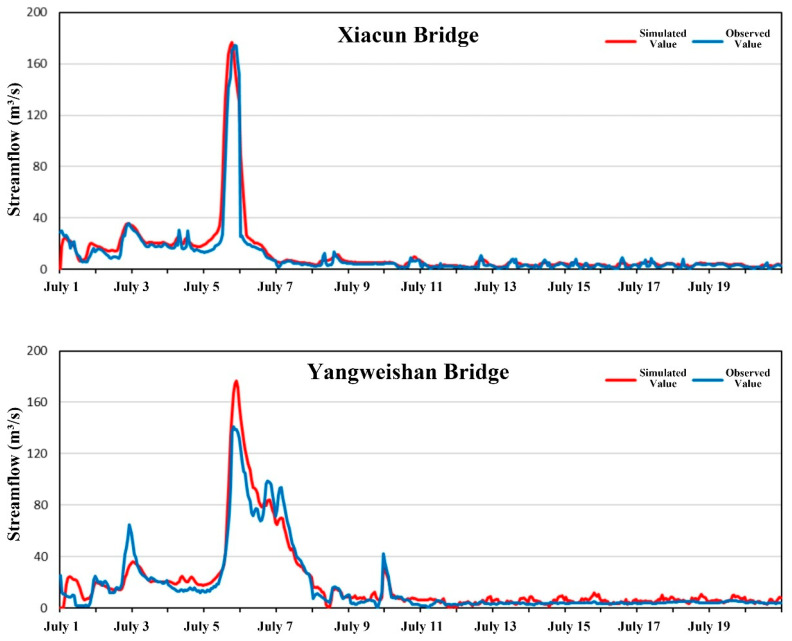
Hydrodynamic model calibration results.

**Figure 4:**
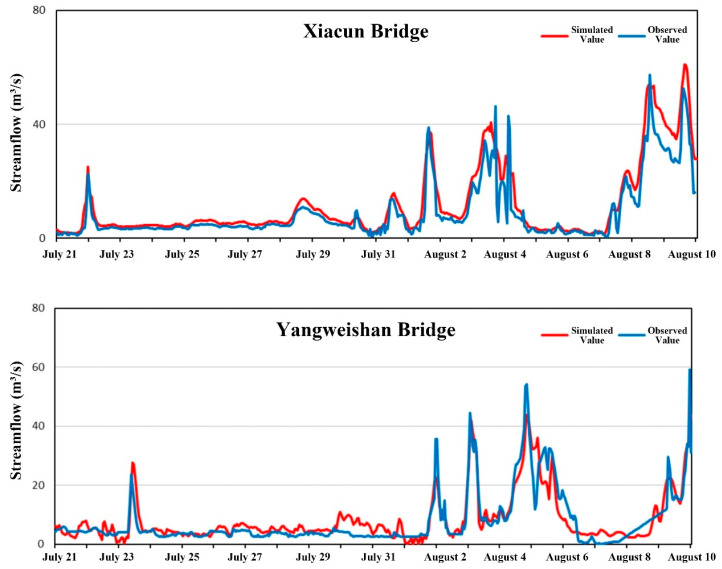
Hydrodynamic model validation results.

Hourly discharge observations from the Xiacun Bridge and Yangweishan Bridge monitoring stations between 1 July and 9 August 2022 were used for hydrodynamic model calibration and validation. Data from 1 to 20 July served for calibration, while data from 21 July to 9 August were reserved for validation. Model performance was evaluated using the Nash–Sutcliffe efficiency (NSE) and visual inspection of simulated versus observed hydrographs (Table 2). The calibration and validation results are shown in Figure 3 and Figure 4. Some discrepancies were noted during the initial simulation period, attributable to model spin-up; agreement improved as the model stabilized, with simulations capturing the overall magnitude and temporal dynamics of the observed discharges. A slight overestimation of the flood peak on 6 July was observed. For the Xiacun Bridge station, NSE values were 0.90 for calibration and 0.79 for validation; corresponding values at Yangweishan Bridge were 0.91 and 0.70. These results indicate that the hydrodynamic model reproduces the flow dynamics of the study reach with acceptable accuracy and is suitable for subsequent analyses.

### 3.2. Water Quality Model Calibration and Validation

**Figure 5:**
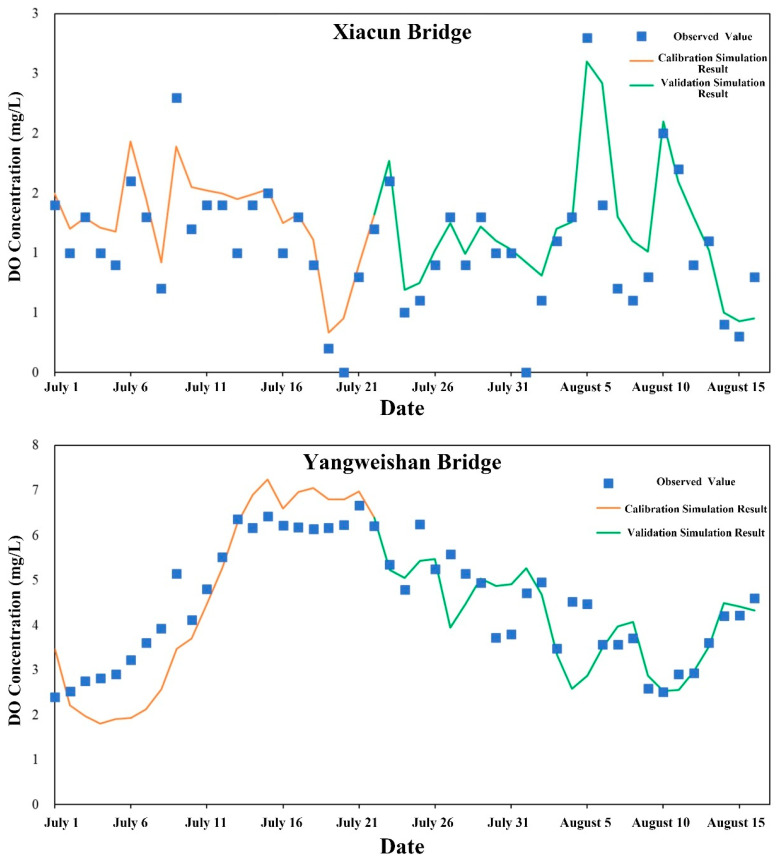
Calibration and validation results for DO simulations from the water quality model.

**Figure 6:**
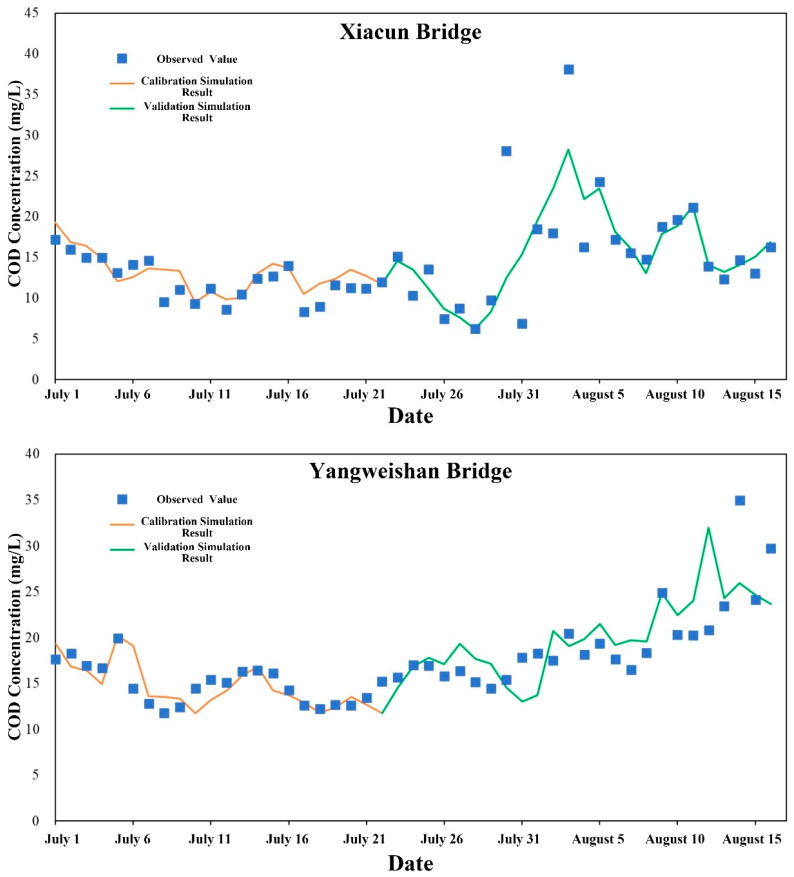
Calibration and validation results for COD simulations from the water quality model.

**Figure 7:**
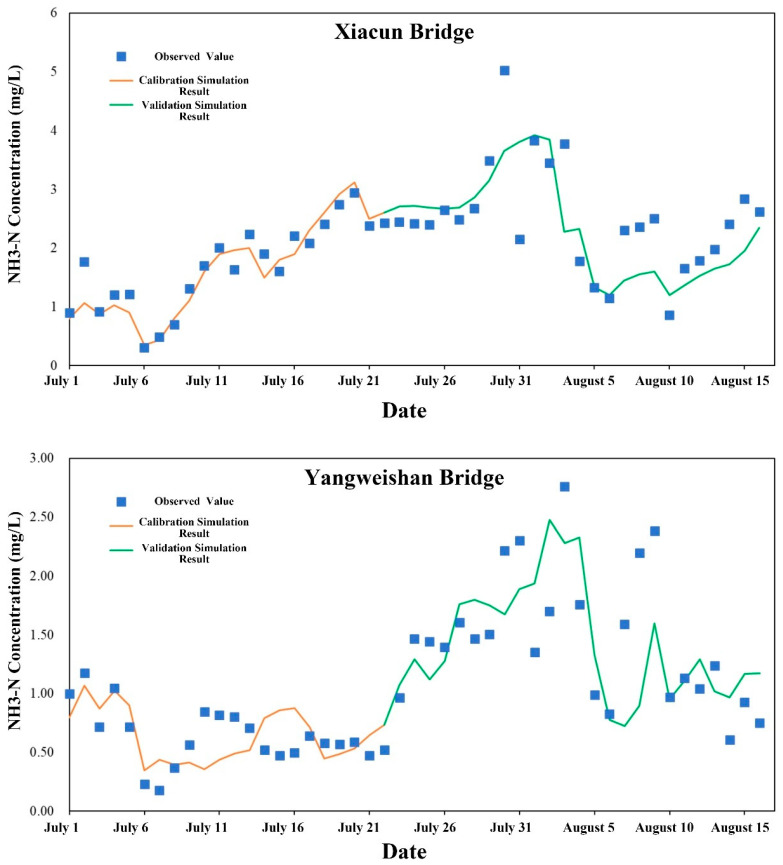
Calibration and validation results for NH_3_-N simulations from the water quality model.

**Figure 8:**
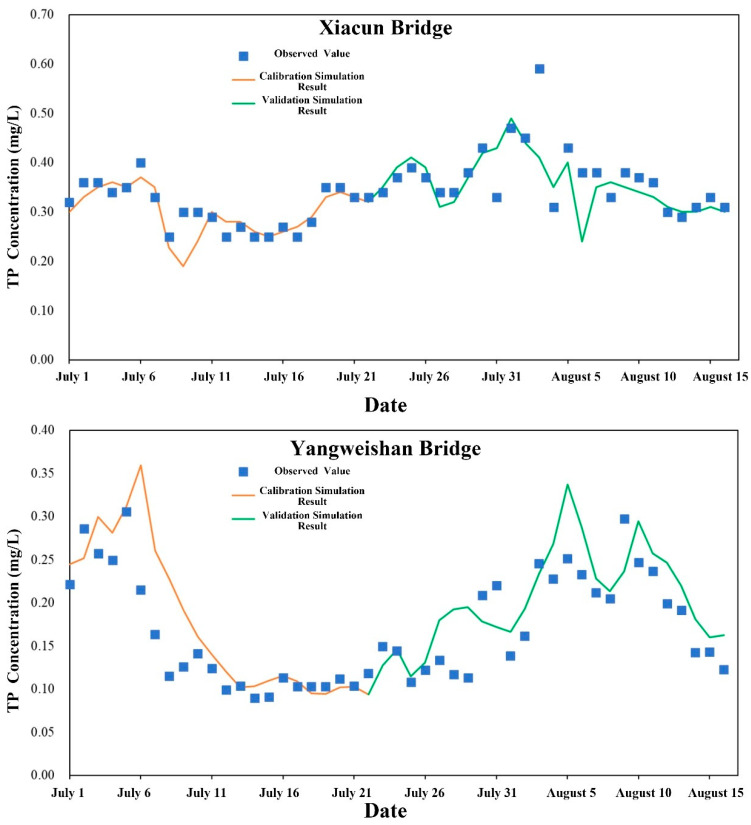
Calibration and validation results for TP simulations from the water quality model.

Daily mean water quality observations from the Xiacun Bridge and Yangweishan Bridge stations between 1 July and 16 August 2022 were employed for water quality model calibration and validation. Data from 1 to 22 July were used for calibration, and data from 23 July to 16 August for validation. Model performance was evaluated using root mean square error (RMSE) and percent bias (PBIAS), complemented by visual comparisons of simulated and observed time series (Table 3 and Table 4). Calibration and validation results for dissolved oxygen (DO), chemical oxygen demand (COD), ammonia nitrogen (NH_3_-N), and total phosphorus (TP) are presented in Figure 5, Figure 6, Figure 7 and Figure 8.

For DO (Figure 5), simulated concentrations at Xiacun Bridge reproduced the observed temporal patterns well, capturing both peak and low values; RMSE and PBIAS were 0.05 and 14.12 for calibration, and 0.07 and 16.56 for validation. At Yangweishan Bridge, larger initial discrepancies were evident, though the overall trend remained consistent, with agreement improving as the simulation stabilized—particularly after 5 August—and RMSE and PBIAS of 0.18 and −3.34 for calibration and 0.15 and −2.82 for validation, indicating generally acceptable DO simulations. Similarly, for COD (Figure 6), model performance during both calibration and validation was excellent. At both stations, simulated patterns during the calibration period closely matched observations; at Xiacun Bridge, RMSE and PBIAS were 0.35 and 7.15 for calibration and 0.90 and −1.18 for validation, while at Yangweishan Bridge the corresponding values were 0.37 and −1.72 for calibration and 0.77 and 2.85 for validation.

The RMSE and PBIAS values at Xiacun Bridge for NH_3_-N (Figure 7) were 0.05 and −2.80 for calibration, 0.14 and −6.67 for validation. At Yangweishan Bridge, values were 0.05 and 0.95 for calibration, 0.10 and −2.53 for validation For TP (Figure 8), model performance was excellent at Xiacun Bridge, and ranged from good (calibration) to excellent (validation) at Yangweishan Bridge. RMSE and PBIAS at Xiacun Bridge were 0.01 and −2.98 for calibration, and 0.01 and −3.34 for validation. At Yangweishan Bridge, corresponding values were 0.01 and 15.97 for calibration, and 0.01 and 12.04 for validation.

Overall, the water quality model successfully reproduced the observed temporal dynamics across all evaluated variables, with simulated trends broadly consistent with measurements. Model performance is inevitably influenced by data quality and uncertainties in point-source discharges, which may contribute to short-term fluctuations in observed concentrations at certain monitoring sites. Despite these limitations, the simulations are generally robust and provide a reliable foundation for subsequent analyses.

## 4. Results

### 4.1. Water Quality Pollution Status in the Puning Section of the Lianjiang River Basin

#### 4.1.1. Water Quality Pollution Status of the Main Stem in the Puning Section

**Figure 9:**
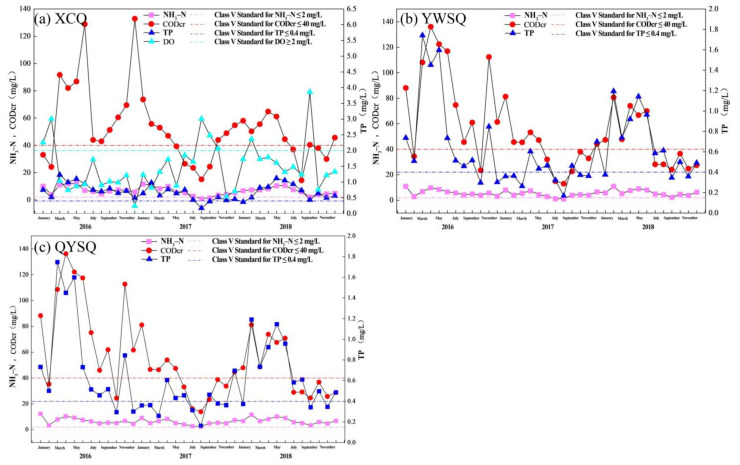
Routine water quality monitoring results for the main stem in the Puning section of the Lianjiang River basin. Note: The dashed line represents the concentration of Class V water quality standard.

During 2016–2018, water quality in the Puning section of the Lianjiang River basin exhibited pronounced temporal variability, with substantial spatial heterogeneity among monitoring sections (Figure 9). At the XCB section, NH_3_-N exceeded the Class V surface water standard for most of the monitoring period, particularly from January 2016 to July 2017, although partial improvement was observed in the second half of 2017 and in early 2018. TP and COD also failed to meet the Class V standard in most months, with notable deterioration during February–March 2016 and May 2018. DO showed strong fluctuations, meeting the standard only in limited periods (e.g., January–February 2016 and August–October 2017). At the YWSB section, NH_3_-N remained relatively stable and was generally below the Class V threshold, whereas TP varied markedly, with a brief period of compliance in mid-2017. COD at this section exceeded the standard in most months, with compliance observed only intermittently. At the QYSB section, NH_3_-N and TP were above the Class V limits for most of the study period, while COD displayed substantial fluctuations, with occasional compliance in selected months, particularly in 2016 and late 2017. Overall, water quality across the Puning section did not meet the Class V standard for most of the monitoring period. Although short-term improvements were observed for specific parameters in some months, these results indicate an urgent need for strengthened water quality management and pollution control in the study area.

#### 4.1.2. Water Quality Pollution Status of Tributaries in the Puning Section

**Figure 10:**
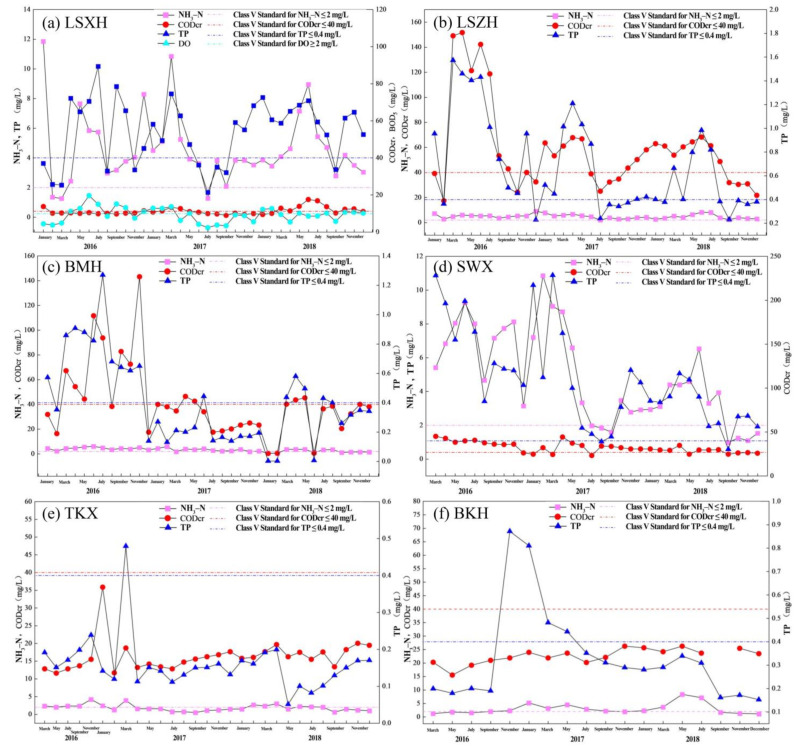
Routine water quality monitoring results for tributaries in the Puning section of the Lianjiang River basin. Note: The dashed line represents the concentration of Class V water quality standard.

During 2016–2018, water quality in the tributaries of the Puning section of the Lianjiang River basin showed pronounced spatial heterogeneity and strong temporal variability (Figure 10). In the LSXR, NH_3_-N exceeded the Class V surface water limit for most of the monitoring period, except for February–April 2016. TP and COD were above the Class V standard in most months, whereas BOD_5_ generally complied with the standard. In the LSZR, NH_3_-N also failed to meet the Class V criterion in most months; TP and COD exhibited substantial fluctuations, with compliance observed in some months during 2017 and 2018. In the BMR, NH_3_-N and TP varied relatively little over time, while COD exceeded the Class V limit in multiple months, with only intermittent compliance. In the SWC, NH_3_-N showed large fluctuations, and TP and COD were largely non-compliant, with COD severely exceeding the standard in several months. By contrast, the TKC exhibited relatively stable NH_3_-N and generally met the Class V standard for TP and COD in most months. In the BKLR, NH_3_-N varied markedly, while TP and COD fluctuated during 2016–2017 and met the Class V criteria in some months. Overall, most tributaries in the Puning section did not meet the Class V surface water standard for substantial portions of the study period, indicating persistent and severe pollution and underscoring the urgent need for strengthened pollution control and water quality management.

### 4.2. Effects of Different Inflow Interception Scenarios on Water Quality Improvement in the Puning Section

Based on an integrated analysis of the drivers of water quality impairment in the Puning section of the Lianjiang River (see Figures S10 and S11, Tables S2–S5 of Text S2 in Supplementary Material), this study explored feasible pathways for improving basin-wide water environment management. The observed pollution pattern is shaped by multiple interacting factors, including socio-economic development, industrial activities, domestic sources, and agricultural practices. Increases in population density and the expansion of night-time light intensity indicate accelerated urbanization, which has raised water demand and, in turn, increased discharges of domestic sewage and commercial wastewater, thereby constituting a major contribution to water quality degradation. Although industrial pollution has shown signs of attenuation, the persistent presence of heavy industry still warrants close attention, particularly with respect to wastewater discharge control. On the agricultural side, excessive fertilizer application and the expansion of livestock and poultry production elevate eutrophication risk, with nitrogen and phosphorus inputs posing a dominant threat to water quality.

Against this background, to better address water quality deterioration, we developed 11 distinct pollutant interception scenarios (Table 5) to evaluate and optimize basin management through inflow control. The scenarios were categorized into single-section interception, two-section combined interception, and three-section combined interception, targeting the inflow points of seven major tributaries within the Lianjiang River basin. The single-section interception scenarios (Scenarios 1–7) focused on individual inflow sections, including Tiande Bridge (TDB) on the BKLR, Dayangmei Bridge (DYMB) on the LSXR, Liudoupu (LDP) on the LSZR, and the inflows associated with the BMR, SWC, XQR, and TKC. Based on tributary-specific inflow conditions and pre-interception water quality levels, corresponding control strategies were designed (see Tables S6 and S7 of Text S3 in Supplementary Material). Overall, these scenarios, implemented as single or combined interception configurations, were intended to reduce basin-scale pollutant loads by controlling discharges at their sources and thereby improve water quality across the river network.

Specifically, the single-section interception scenarios (e.g., Scenarios 1–7) (Figures S12–S18) achieved measurable reductions in pollutant concentrations, but their effectiveness varied substantially among sections and the overall load reduction remained limited. For instance, Scenario 1 (interception at TDB on the BKLR) produced marked decreases in COD, NH_3_-N, and TP at downstream sections, with particularly strong performance for nitrogen control; COD, NH_3_-N, and TP decreased by 57.81%, 65.69%, and 41.39%, respectively (Figure S12). In contrast, Scenario 2 (interception at DYM on the LSXR) was more effective for reducing COD and TP, but showed weaker performance for nitrogen abatement, with overall reductions generally lower than those achieved under Scenario 1.

By comparison, the combined two-section scenarios (Scenarios 8–10) (Figures S25–S27) and the three-section scenario (Scenario 11) (Figure S22) exhibited a clear additive (synergistic) effect, enabling more comprehensive reductions in basin pollution, and demonstrating stronger integrated control of COD, NH_3_-N, and TP. Scenario 8 (interception at TDB and DYM) achieved more than 70% reductions in COD and nitrogen-related indicators across the three monitoring sections, while TP was reduced by approximately 65%, highlighting its advantage for multi-parameter co-control. Scenario 9 (interception at TDB and LDP) also delivered substantial improvements, with NH_3_-N reductions of approximately 80%. As the best-performing multi-source option, Scenario 11 (simultaneous interception at TDB, DYMB, and LDP) resulted in near-complete COD abatement and pronounced decreases in downstream concentrations; COD, NH_3_-N, and TP were reduced by 85.07%, 97.56%, and 92.08%, respectively, demonstrating strong synergistic benefits of coordinated multi-source interception and substantially improving water quality at the basin scale.

### 4.3. Water Quality Improvements Under Different Key Engineering Intervention Scenarios in the Puning Section

To quantify the water quality benefits associated with different key engineering interventions (e.g., wastewater purification plants), four implementation scenarios were designed (Table S8). Scenario 1 used the daily mean effluent concentrations from the two existing facilities in 2022 as the baseline. Scenario 2 represented operation of the Puning municipal wastewater purification plant only, whereas Scenario 3 considered operation of the Huaxi Village integrated drainage channel treatment facility only. Scenario 4 further assumed the addition of a new treatment facility at the TDB on the BKLR, parameterized based on the treatment capacity of the Puning plant; the corresponding effluent concentration was derived after accounting for the calculated discharge at this section, and both existing facilities were operated simultaneously. Focusing on COD, NH_3_-N, and TP, the four scenarios were compared to elucidate how pollution load reductions under different engineering configurations translate into integrated water quality responses in the river network.

**Figure 11:**
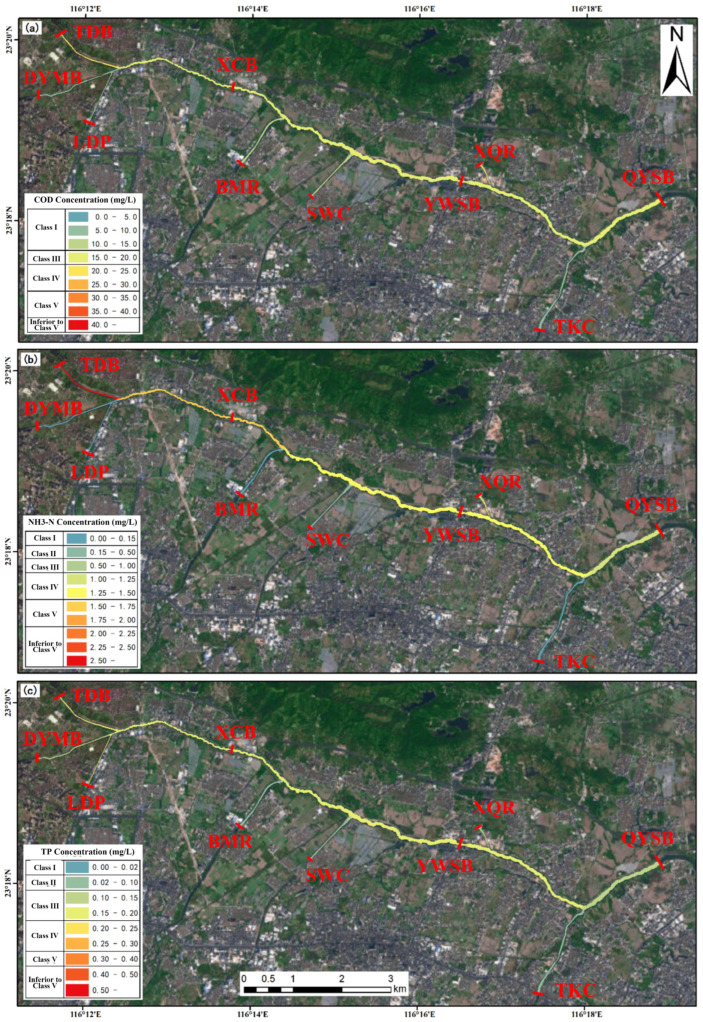
Spatial distribution of pollutant loads under Scenario 1 (joint operation of the Puning wastewater purification plant on the LSXR and the Huaxi Village drainage channel treatment facility on the LSZR). (**a**) COD; (**b**) NH_3_-N; (**c**) TP.

Specifically, the four engineering scenarios differed markedly in their pollutant reduction performance, highlighting the advantage of integrated interventions for water quality improvement. Scenario 1 (joint operation of the Puning wastewater purification plant on the LSXR and the Huaxi Village drainage channel treatment facility on the LSZR) delivered the most favorable overall performance (Figure 11). At the three monitoring sections (XCB, YWSB, and DYMB), COD, NH_3_-N, and TP concentrations decreased by 26.68%, 32.93%, and 30.23%; 22.14%, 33.15%, and 27.30%; and 21.79%, 31.66%, and 27.22%, respectively. By combining the municipal purification plant with the drainage channel treatment facility, this scenario produced consistent downstream water quality improvements, with particularly strong NH_3_-N abatement (mean reduction of approximately 32%). Overall, Scenario 1 showed robust pollutant load reduction with relatively balanced improvements across sections, indicating its effectiveness for basin-scale water environment management.

**Figure 12:**
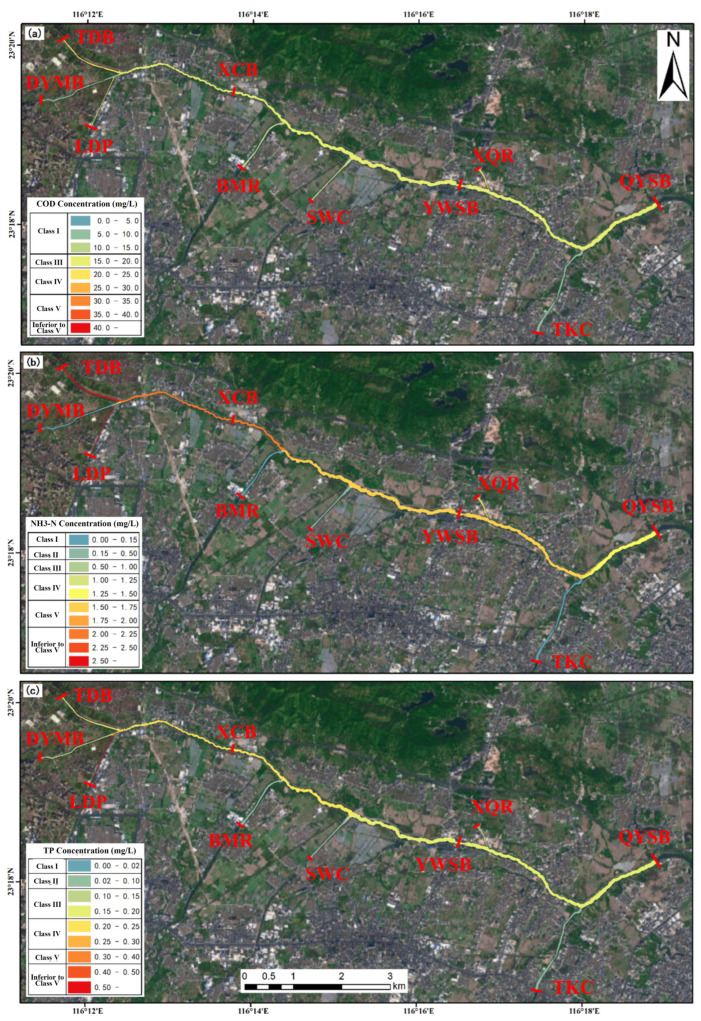
Spatial distribution of pollutant loads under Scenario 2 (operation of the Puning wastewater purification plant only): (**a**) COD; (**b**) NH_3_-N; (**c**) TP.

**Figure 13:**
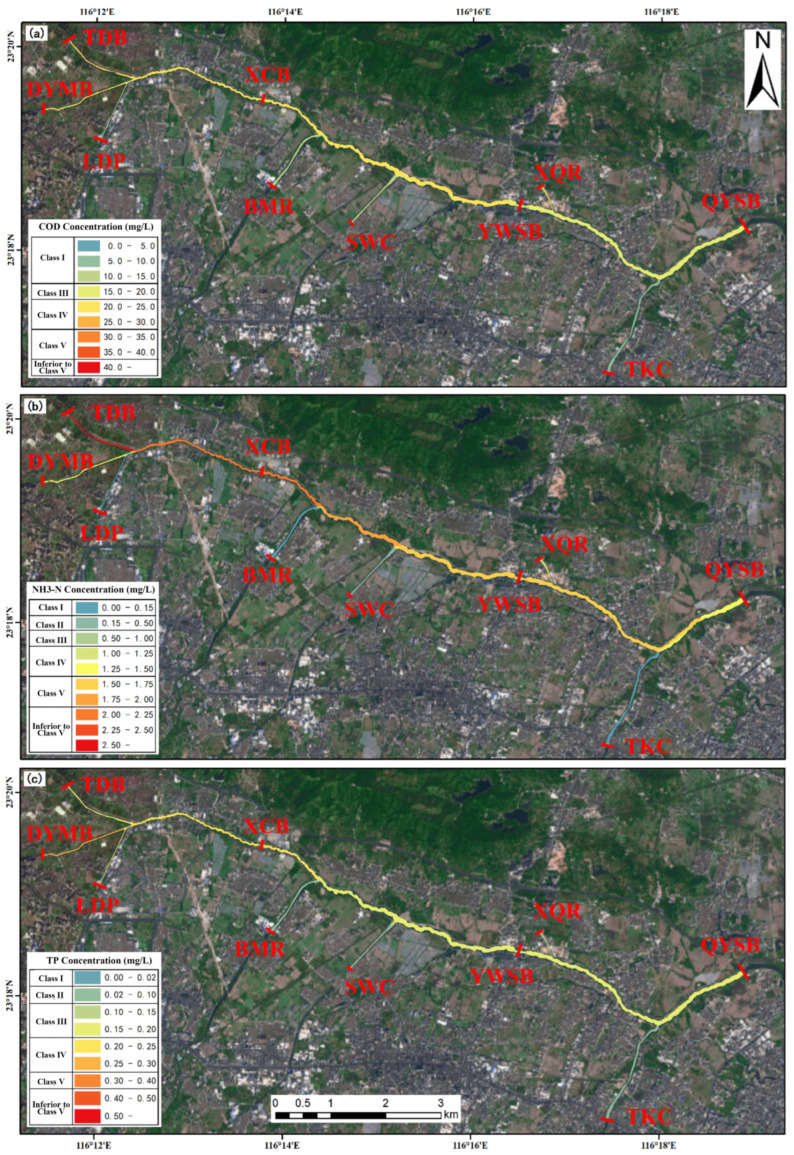
Spatial distribution of pollutant loads under Scenario 3 (operation of the Huaxi Village integrated drainage channel treatment facility only). (**a**) COD; (**b**) NH_3_-N; (**c**) TP.

**Figure 14:**
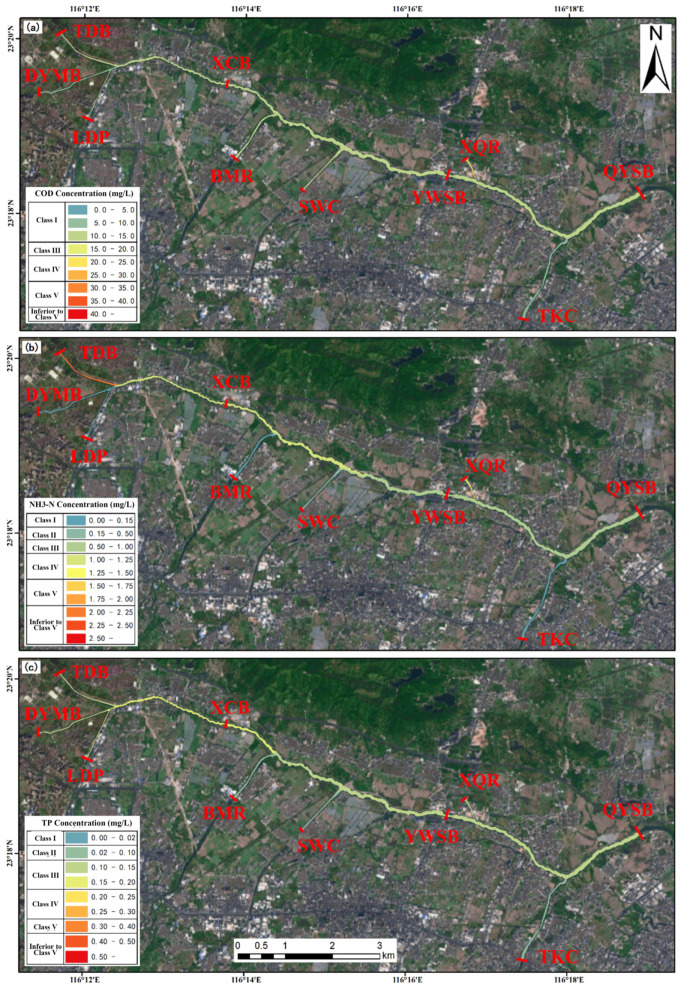
Spatial distribution of pollutant loads under Scenario 4 (additional wastewater treatment plant constructed at the TDB section of the BKLR). (**a**) COD; (**b**) NH_3_-N; (**c**) TP.

In contrast, Scenario 2 (operation of the Puning wastewater purification plant only) performed relatively well in COD abatement, but was less effective for nitrogen and phosphorus control (Figure 12). Across the three downstream monitoring sections, COD decreased by 18.13–22.01%, whereas NH_3_-N and TP showed more modest reductions (10–18%), indicating that a single facility provides limited benefits, particularly for nutrient mitigation. Scenario 3 (operation of the Huaxi Village integrated drainage channel treatment facility only) showed comparatively stronger performance for phosphorus reduction, with TP decreasing by more than 16% (Figure 13). However, improvements in COD and NH_3_-N were limited; notably, COD at the YWSB section decreased by only 4.68%. This scenario may therefore be more suitable for localized water quality improvement or targeted phosphorus control, but its overall effectiveness was inferior to that achieved under combined operation. Scenario 4 (addition of a new wastewater treatment plant based on the capacity of the Puning plant, with simultaneous operation of the two existing facilities) exhibited a pronounced synergistic effect across all three pollutant groups. In particular, NH_3_-N decreased by approximately 50% at all three monitoring sections, while COD and TP were reduced by more than 40% and approximately 40%, respectively, demonstrating the clear advantage of integrated engineering interventions (Figure 14). Spatially, Scenario 4 delivered relatively balanced improvements among sections and substantially outperformed the single-facility scenarios in terms of overall pollutant load reduction.

### 4.4. Water Quality Improvements Under Integrated Water Environment Management Scenarios in the Puning Section

Six representative scenarios were developed to evaluate how different management measures would affect water quality in the Lianjiang River basin (Table 6). Scenario 1 represents centralized industrial pollution control, in which a dedicated wastewater treatment plant for the textile-dyeing industrial cluster is constructed to collect industrial effluents and ensure compliant discharge. Scenario 2 focuses on urban–rural sewer network upgrading, reducing riverine pollutant inputs by increasing sewer collection coverage. Scenario 3 evaluates the construction of new wastewater treatment plants (e.g., at Yinggeshan, Meitang Town, and Yunluo Town), and simulates the additive water quality benefits associated with their joint operation. Scenario 4 represents ecological water replenishment, whereby ecological baseflow is supplemented for the LSXR and LSZR to increase dissolved oxygen and strengthen natural self-purification. Scenario 5 examines channel dredging to reduce internal loading by removing contaminated sediments and thereby improve water quality. Scenario 6 considers riparian ecological buffer zones, in which vegetated buffers are established to enhance self-purification and reduce pollutant inputs from surface runoff.

The results show that the six scenarios differed substantially in their effectiveness, and each measure exhibited distinct strengths and limitations in pollutant abatement. Under the centralized industrial control scenario (Scenario 1), construction of the textile-dyeing industrial treatment center markedly reduced the industrial load at the DYMB section of the LSXR (Figure S23). After implementation, COD, NH_3_-N, and TP decreased consistently at the downstream sections (XCB, YWSB, and QYSB), with mean reductions of approximately 7% for COD and approximately 32% and approximately 30% for NH_3_-N and TP, respectively. This indicates strong effectiveness for nitrogen mitigation, with additional benefits for phosphorus and organic pollution control. The sewer network upgrading scenario (Scenario 2), which increased sewer coverage to 90%, effectively reduced riverine pollutant inputs (Figure S24). Across the three monitoring sections, COD, NH_3_-N, and TP decreased by approximately 15%, demonstrating stable and balanced co-control of carbonaceous, nitrogenous, and phosphorus pollutants at the basin scale. The new wastewater treatment plant scenario (Scenario 3) simulated the commissioning of multiple wastewater purification plants (Figures S25 and S26). When only the Yinggeshan wastewater purification plant was operated, COD and NH_3_-N decreased by approximately 2% and approximately 2.5%, respectively, whereas TP increased, suggesting limited benefits from a single new facility. Under the joint operation of five wastewater purification plants, COD and NH_3_-N decreased slightly further (to approximately 3%), but TP reduction remained marginal and small TP increases were still observed at some sections. Overall, new wastewater purification plants provided measurable control of COD and NH_3_-N, but their effectiveness for TP abatement was limited.

The ecological water replenishment scenario (Scenario 4) substantially increased DO, particularly in the upstream reach, where DO improved by 27.47%. COD and NH_3_-N also decreased (by approximately 8% and approximately 20%, respectively), reflecting enhanced self-purification associated with increased ecological baseflow (Figure S27). However, TP increased at some sections, indicating limited capability for phosphorus control. This may be because ecological water replenishment can disturb sediments and release internal phosphorus, while variations in flow velocity affect phosphorus sedimentation and resuspension. The channel dredging scenario (Scenario 5) reduced internal loading and was especially effective for phosphorus removal: TP declined markedly at all monitoring sections, with a mean reduction of approximately 17% (Figure S28). By contrast, NH_3_-N reductions were minor, implying limited effectiveness for nitrogen mitigation. Finally, the riparian ecological buffer scenario (Scenario 6), implemented along the Baima River and SWC, resulted in modest reductions at mid- to downstream sections and showed relatively consistent performance for TP control (mean reduction of approximately 1.3%) (Figure S29). However, reductions in COD and NH_3_-N were small, suggesting that the buffer intervention alone provides limited benefits for carbon and nitrogen mitigation.

## 5. Discussion

### 5.1. Comparative Assessment of Management Scenarios

Based on the pollutant reduction performance of the 11 interception scenarios (Table S9), clear differences were observed in the abatement of nitrogen and phosphorus pollutants, with discernible spatial patterns. The single-section interception scenarios (Scenarios 1–7) provided limited downstream improvement and showed evident constraints in nitrogen control. Among them, Scenario 1 (interception at TDB on the BKLR) performed relatively well, reducing COD, NH_3_-N, and TP by 57.81%, 65.69%, and 41.39%, respectively. The two-source combined interception scenarios (Scenarios 8–10) exhibited pronounced additive benefits. In particular, Scenarios 8 and 9 achieved reductions in more than 70% for COD and NH_3_-N at the TDB section, while TP decreased by nearly 65%. Scenario 10 delivered slightly lower reductions for COD and NH_3_-N than Scenarios 8–9, but still performed well for TP control. The multi-source combined interception scenario (Scenario 11) was identified as the best-performing option. By jointly intercepting inputs at TDB on the BKLR, DYMB on the LSXR, and LDP on the LSZR, Scenario 11 achieved highly efficient reductions across monitoring sections, with COD, NH_3_-N, and TP reduced by 100%, 97.56%, and 92.08%, respectively. This scenario substantially improved basin-wide water quality and clearly demonstrated the synergistic benefits of coordinated multi-source interception. Overall, Scenario 11 outperformed all alternatives and represents the most effective strategy for water environment management in the Lianjiang River basin.

Based on the pollutant reduction performance of the four engineering implementation scenarios (Table S10), clear differences were observed in the reductions in COD, NH_3_-N, and TP, and combined engineering configurations consistently outperformed single-facility operation. The single-facility scenarios (Scenarios 2 and 3) improved water quality, but the magnitude of reduction was relatively limited. Scenario 2, centered on the municipal wastewater purification plant, achieved comparatively better COD abatement but provided limited control of nitrogen and phosphorus. Scenario 3, centered on the integrated drainage channel treatment facility, performed better for phosphorus mitigation but showed weak COD reduction. For both single-facility scenarios, improvements were mainly confined to upstream sections, with limited benefits propagated to downstream locations. In contrast, the combined engineering scenarios (Scenarios 1 and 4) produced substantially greater improvements, particularly when the wastewater purification plant and the drainage channel facility were jointly implemented in the LSXR and LSZR. Scenario 1 yielded relatively balanced reductions, with COD, NH_3_-N, and TP decreasing by approximately 22–26%, 32%, and 27–30%, respectively. Scenario 4 further enhanced pollutant removal, reducing COD, NH_3_-N, and TP by 49.37%, 50.82%, and 42.39%, respectively, and delivered stronger spatial consistency and stability across monitoring sections. Overall, the combined engineering configurations not only achieved greater basin-wide pollutant reduction than the single-facility options, but also produced more spatially uniform improvements, indicating superior practicality and implementability.

Based on the pollutant reduction performance of the seven integrated management options evaluated across the six representative scenarios (Table S11), the results indicate clear differences in the control of COD, NH_3_-N, and TP, with integrated interventions generally outperforming single-measure options. Scenario 2 (urban–rural sewer network upgrading) delivered relatively stable reductions, with mean decreases of approximately 15% for COD, NH_3_-N, and TP. Scenario 4 (ecological water replenishment) was particularly effective for improving DO and reducing NH_3_-N achieving more than 25% improvement at upstream sections and more than 15% at downstream sections. Scenario 5 (channel dredging) showed stronger effectiveness for phosphorus control, with TP reduced by an average of approximately 17% across the three sections. Overall, these findings indicate that integrated management, by jointly implementing different measures, can markedly enhance water quality improvement; in particular, sewer network upgrading and ecological water replenishment showed consistently strong performance across multiple water quality indicators, providing effective technical support for regional water environment management.

### 5.2. Integrated Management Evaluation and Recommendations

For water quality management in the Lianjiang River basin, the results indicate that single-section interception measures can achieve noticeable improvements at upstream locations, but their effectiveness in controlling pollution at downstream sections is limited. In contrast, the two-source combined interception configurations maintained more stable performance in the mid- to downstream reaches. In particular, options involving interception at the TDB on the BKLR enabled more balanced improvements between upstream and downstream sections. Further implementation of multi-source combined interception substantially reduced inter-section differences in pollutant reduction and promoted basin-wide water quality improvement. Overall, the evaluation suggests that single interception measures may be suitable for localized control or low-intensity pollution conditions, but are relatively less effective for nutrient (nitrogen and phosphorus) mitigation. Two-source combined interception can effectively reduce COD and TP and shows clear advantages for broader pollution control. The multi-source combined interception configuration (Scenario 11) delivered the best overall performance, and is particularly suitable for improving water quality in downstream reaches, representing the preferred strategy for basin-scale restoration. In addition, joint implementation of different management measures can enhance treatment performance through synergistic operation, with clear benefits for controlling COD, NH_3_-N, TP, and improving DO. Among the integrated management options, Scenarios 2, 4, and 5 were especially effective in achieving systematic basin-wide improvement, providing robust strategic support for river water environment management.

This perspective is corroborated by findings from other studies. Yuan et al. [35] conducted simulation analyses on conventional pollution indicators in the Nandu River, where they modeled the installation of sluice gates on three tributaries flowing into the mainstem. Their research focused on mitigating pollution in the main river channel by controlling the opening and closing of these gates to reduce contaminant levels. Similarly, Ji et al. [36] simulated comprehensive pollution remediation strategies for COD, ammonia nitrogen, and total phosphorus in the Beijing sub-center. Their study applied Low Impact Development (LID) measures to control urban non-point source pollution, Best Management Practices (BMPs) to manage agricultural non-point sources, and a combination of both approaches. The results demonstrated that these integrated measures achieved approximately a 30% reduction in multiple pollutants within the area. These studies collectively demonstrate that approaches focusing on a single control unit or a single treatment method can only address pollutants from specific areas or sectors. Achieving overall water quality improvement in a region necessitates a comprehensive remediation strategy that integrates multiple processes, diverse measures, and cross-sectoral coordination.

### 5.3. The Application Prospects of Model Transferability

The Lianjiang River Basin in Puning is located in a region characterized by intensive human activities. Therefore, some scenario settings are based on realistic assumptions according to actual land use conditions, such as the configuration of wastewater treatment plants. When applied to other watersheds, multi-scenario simulations can be conducted by configuring corresponding pollution control measures in areas with strong human activities, thereby providing guidance for engineering practices. The MIKE 21 model is versatile and supports local parameter calibration. All scenarios are configured using this software (DHI MIKE 21 2014 (MIKE Zero platform)). In data-scarce regions, parameter estimation can be improved by integrating remote sensing and statistical data to enhance the model’s applicability. Therefore, we believe that the model and methodology adopted in this study can be effectively replicated and extended to other regions.

## 6. Conclusions

This study combined comprehensive field assessment and numerical modeling to evaluate water quality management options for the Puning section of the Lianjiang River basin and to provide a technical basis for basin remediation. The results indicate that water quality in the study reach is jointly affected by point source and non-point source inputs, with domestic sewage, industrial wastewater, and agricultural non-point source as the dominant contributors. Most monitoring sections failed to meet the Class V surface water standard, particularly with respect to COD, NH_3_-N, and TP. Using the MIKE 21 coupled hydrodynamic and water quality model, 22 management scenarios were simulated. The modeling results demonstrate that multi source interception configurations, especially those integrating control of multiple key pollution sources, achieved the greatest reductions in pollutant concentrations. Notably, the combined multi source interception scenario produced substantial abatement of COD, NH_3_-N, and TP, with maximum reductions of up to 97% for NH_3_-N and TP. Overall, the findings highlight the importance of integrated strategies for basins with complex pollution sources, indicating that a portfolio of measures is required to achieve effective water quality improvement. Such measures include interception, ecological restoration, and upgrades to wastewater treatment infrastructure. The outcomes provide technical support and a reference basis for water environment management in the Lianjiang River basin and offer transferable and sustainable insights for water quality management in similar regions.

## Figures and Tables

**Table 1:** The criteria for evaluating the performance of the model.

	NSE	PBIAS (%)	RMSE
Excellent	0.75 < NSE ≤ 1.00	PBIAS < ±10	0.00 < RMSE ≤ 1.00
Good	0.65 < NSE ≤ 0.75	±10 < PBIAS ≤ ±15	1.00 < RMSE ≤ 10.00
Acceptable	0.50 < NSE ≤ 0.65	±15 < PBIAS ≤ ±25	
Poor	NSE ≤ 0.50	PBIAS ≥ ±25	RMSE ≥ 10.00

**Table 2:** The NSE evaluation results of the hydrodynamic model.

NSE	Calibration	Validation
Xiacun Bridge	0.90	0.79
Yangweishan Bridge	0.91	0.70

**Table 3:** The RMSE evaluation results of the water quality model.

RMSE	NH_3_-N	COD	TP	DO
Xiacun Bridge				
calibration	0.05	0.35	0.01	0.05
validation	0.14	0.90	0.01	0.07
Yangweishan Bridge				
calibration	0.05	0.37	0.01	0.18
validation	0.10	0.77	0.01	0.15

**Table 4:** The PBIAS evaluation results of the water quality model.

PBIAS	NH_3_-N	COD	TP	DO
Xiacun Bridge				
calibration	−2.80	7.15	−2.98	14.12
validation	−6.67	−1.18	−3.34	16.56
Yangweishan Bridge				
calibration	0.95	−1.72	15.97	−3.34
validation	−2.53	2.85	12.04	−2.82

**Table 5:** Configuration of the 11 inflow interception scenarios.

Pollution Control Scenarios		Pollutant Interception Section
Single section	Scenario 1	TDB on the BKLR
	Scenario 2	DYMB on the LSXR
	Scenario 3	LDP on the LSZR
	Scenario 4	BMR
	Scenario 5	SWC
	Scenario 6	XQR
	Scenario 7	TKC
Two sections	Scenario 8	TDB on the BKLR, DYMB on the LSXR
	Scenario 9	TDB on the BKLR, LDP on the LSZR
	Scenario 10	DYMB on the LSXR, LDP on the LSZR
Three sections	Scenario 11	TDB on the BKLR, DYM on the LSXR, LDP on the LSZR

**Table 6:** Configuration of integrated management implementation scenarios (mg/L).

Scenarios	Pollutants	TDB	DYMB	LDP	BMR	SWC	XQR	TKC
Scenario 1	COD	24.20	16.44	22.80	12.58	13.10	21.32	9.79
	NH_3_-N	2.96	1.10	3.09	0.13	0.29	1.28	0.13
	TP	0.20	0.23	0.61	0.04	0.09	0.13	0.08
Scenario 2	COD	20.57	18.85	19.38	10.69	11.14	18.12	8.33
	NH_3_-N	2.52	1.26	2.63	0.11	0.25	1.09	0.11
	TP	0.17	0.21	0.52	0.04	0.08	0.11	0.07
Scenario 3–1	COD	23.63	22.18	22.80	12.58	13.10	21.32	9.79
	NH_3_-N	2.85	1.48	3.09	0.13	0.29	1.28	0.13
	TP	0.20	0.25	0.61	0.04	0.09	0.13	0.08
Scenario 3–2	COD	23.63	21.62	22.80	12.58	13.10	19.33	9.79
	NH_3_-N	2.85	1.41	3.09	0.13	0.29	0.94	0.13
	TP	0.20	0.25	0.61	0.04	0.09	0.16	0.08
Scenario 4	Flow (m^3^/s)	3.49	3.10	1.86	1.20	0.42	0.18	0.83
	COD	24.20	18.68	17.22	12.58	13.10	21.32	9.79
	DO	2.71	3.83	3.13	5.68	4.68	4.10	5.50
	NH_3_-N	2.96	0.97	1.42	0.13	0.29	1.28	0.13
	TP	0.20	0.24	0.40	0.04	0.09	0.13	0.08
Scenario 5	COD	24.20	22.18	22.80	12.58	13.10	21.32	9.79
	NH_3_-N	2.95	1.48	3.06	0.11	0.14	0.91	0.02
	TP	0.20	0.25	0.60	0.04	0.05	0.04	0.05
Scenario 6	COD	24.20	22.18	22.80	10.69	11.14	21.32	9.79
	NH_3_-N	2.96	1.48	3.09	0.09	0.20	1.28	0.13
	TP	0.20	0.25	0.61	0.03	0.06	0.13	0.08

## Data Availability

The data that support the findings of this study are available from the corresponding author upon reasonable request.

## Supplementary Materials

The following supporting information can be downloaded at: https://www.mdpi.com/article/10.3390/toxics14030216/s1, Text S1: MIKE 21 model setup; Text S2: Preliminary diagnosis of water pollution sources; Text S3: Effects of different inflow interception scenarios on water quality improvement in the Puning reach of the Lianjiang River basin; Text S4: Water quality improvements under different key engineering intervention scenarios in the Puning reach of the Lianjiang River basin; Text S5: Water quality improvements under integrated water environment management scenarios in the Puning reach of Lianjiang River basin; Text S6: Comparative assessment of management scenarios; Figure S1: Distribution of unmanned vessel bathymetry points and DEM in the Lianjiang River basin; Figure S2: Topographic grid map of the Lianjiang River basin; Figure S3: Grid topographic map of the Lianjiang River Basin; Figure S4: Spatial variation of bottom friction in the Lianjiang River basin; Figure S5: Spatial variation of initial water levels for the hydrodynamic module; Figure S6: Spatial variation of initial DO concentrations; Figure S7: Spatial variation of initial COD concentrations; Figure S8: Spatial variation of initial NH_3_-N concentrations; Figure S9: Spatial variation of initial TP concentrations; Figure S10: Population density in the Puning reach of the Lianjiang River basin; Figure S11: Distribution of night-time lights in the Puning reach of the Lianjiang River basin from 2014 to 2022; Figure S12: Spatial distribution of pollutant loads under Scenario 1 (interception at the TDB section on the BKLR); (a) COD; (b) NH_3_-N; (c) TP; Figure S13: Spatial distribution of pollutant loads under Scenario 2 (interception at the DYMB section on the LSXR); (a) COD; (b) NH_3_-N; (c) TP; Figure S14: Spatial distribution of pollutant loads under Scenario 3 (interception at the LDP section on the LSZR); (a) COD; (b) NH_3_-N; (c) TP; Figure S15: Spatial distribution of pollutant loads under Scenario 4 (interception at the BMR section); (a) COD; (b) NH_3_-N; (c) TP; Figure S16: Spatial distribution of pollutant loads under Scenario 5 (interception at the SWC section); (a) COD; (b) NH_3_-N; (c) TP; Figure S17: Spatial distribution of pollutant loads under Scenario 6 (interception at the XQR section); (a) COD; (b) NH_3_-N; (c) TP; Figure S18: Spatial distribution of pollutant loads under Scenario 7 (interception at the TKC section); (a) COD; (b) NH_3_-N; (c) TP; Figure S19: Spatial distribution of pollutant loads under Scheme 8 (simultaneous interception at the TDB section of BKLR and the DYMB section of the LSXR River): (a) COD; (b) NH_3_–N; (c) TP; Figure S20: Spatial distribution of pollutant loads under Scenario 9 (simultaneous interception at TDB on the BKLR and LDP on the LSZR): (a) COD; (b) NH_3_-N; (c) TP; Figure S21: Spatial distribution of pollutant loads under Scenario 10 (simultaneous interception at DYMB on the LSXR and LDP on the LSZR): (a) COD; (b) NH_3_-N; (c) TP; Figure S22: Spatial distribution of pollutant loads under Scenario 11 (simultaneous interception at TDB on the BKLR, DYMB on the LSXR, and LDP on the LSZR): (a) COD; (b) NH_3_-N; (c) TP; Figure S23: Spatial distribution of pollutant loads under Scenario 1 (construction of a centralized textile-dyeing wastewater treatment center in the LSXR): (a) COD; (b) NH_3_-N; (c) TP; Figure S24: Spatial distribution of pollutant loads under Scenario 2 (sewer network upgrading in the urban reach of Puning City, Lianjiang River Basin): (a) COD; (b) NH_3_-N; (c) TP; Figure S25: Spatial distribution of pollutant loads under Scenario 3–1 (construction of a new wastewater treatment plant in the urban reach of Puning City, Lianjiang River Basin): (a) COD; (b) NH_3_-N; (c) TP; Figure S26: Spatial distribution of pollutant loads under Scenario 3–2 (construction of a new wastewater treatment plant in the urban reach of Puning City, Lianjiang River Basin): (a) COD; (b) NH_3_–N; (c) TP; Figure S27: Spatial distribution of pollutant loads under Scenario 4 (ecological water replenishment in the LSXR and the LSZR): (a) COD; (b) DO; (c) NH_3_NH_3_–N; (d) TP; Figure S28: Spatial distribution of pollutant loads under Scenario 5 (river dredging in the urban reach of Puning City, Lianjiang River Basin): (a) NH_3_-N; (b) TP; Figure S29: Spatial distribution of pollutant loads under Scenario 6 (construction of ecological buffer strips along the BMR and the SWC): (a) COD; (b) NH_3_–N; (c) TP; Table S1; Model parameter settings; Table S2: Puning city’s annual resident population and national economic data from 2014 to 2020; Table S3: Number of industrial enterprises in Puning city from 2014 to 2022; Table S4: Number of livestock and poultry in Puning city from 2014 to 2022; Table S5: Fertilizer application in Puning city from 2014 to 2022; Table S6: Inflow conditions at the monitoring sections; Table S7: Initial water quality conditions at the monitoring sections before interception (mg/L); Table S8: Scenario settings for four key engineering interventions (mg/L); Table S9: Pollutant reduction efficiencies of 11 interception-based management scenarios (unit: %); Table S10: Pollutant reduction efficiencies of four engineering implementation schemes (unit: %); Table S11: Pollutant reduction efficiencies of four engineering implementation schemes (unit: %).
