# Supplementary material for: Modeling and Evaluating Integrated Pollution Control Measures in Rivers: A Case Study of the Lianjiang River Basin
Source: Toxics. 2026 Mar 1;14(3):216. doi: 10.3390/toxics14030216 (PMC13029882; doi:10.3390/toxics14030216)
Supplement: Supplementary file 1 [file toxics-14-00216-s001.zip › toxics-4147805-supplementary.pdf]

Supporting Information for

Modeling and Evaluating Integrated Pollution Control Measures in Rivers: A Case Study of the Lianjiang River Basin

Jinxi Zheng[12], Yongyou Hu[1], Wenqin Xu[3], Shewei Yang[3], Xiangzhuan Zeng[4], Youshun Guo[3], Zhenjiang Yu[3], Jianhua Cheng[1*]

1. Key Lab Pollut Control & Ecosyst Restorat Ind Clus, Minist Educ, College of Environment and Energy, South China University of Technology, Guangzhou 510006, China
2. Guangdong Yifang Environmental Protection Technology Company Limited
3. Guangdong Provincial Academy of Environmental Science
4. Guangdong Enweile Environmental Technological Company Limited

Corresponding author: jhcheng@scut.edu.cn

Text S1 MIKE 21 model setup

1. Terrain Mesh Creation

The quality of the terrain mesh plays a crucial role in determining the simulation accuracy of the MIKE 21 model. In this study, an irregular triangular mesh was used, and the mesh was generated using the Surface Water Modeling System (SMS) grid generation tool.

2. Computational Region Extraction

The first step in constructing the coupled hydrodynamic-water quality model is to extract the boundary data for the target study area, which covers the Puning reach of the Lianjiang River basin, including the TDB on the BKLR, the DYMB on the LSXR, the LDP on the LSZR, and the QYSB, as well as seven tributary inflows. The study area boundary was delineated by loading the Tian Di Tu base map in ArcGIS, depicting the surrounding embankments and defining the external boundary of the study region. The water surface area along the river boundary was then outlined to serve as the internal boundary of the model (Fig. S1).

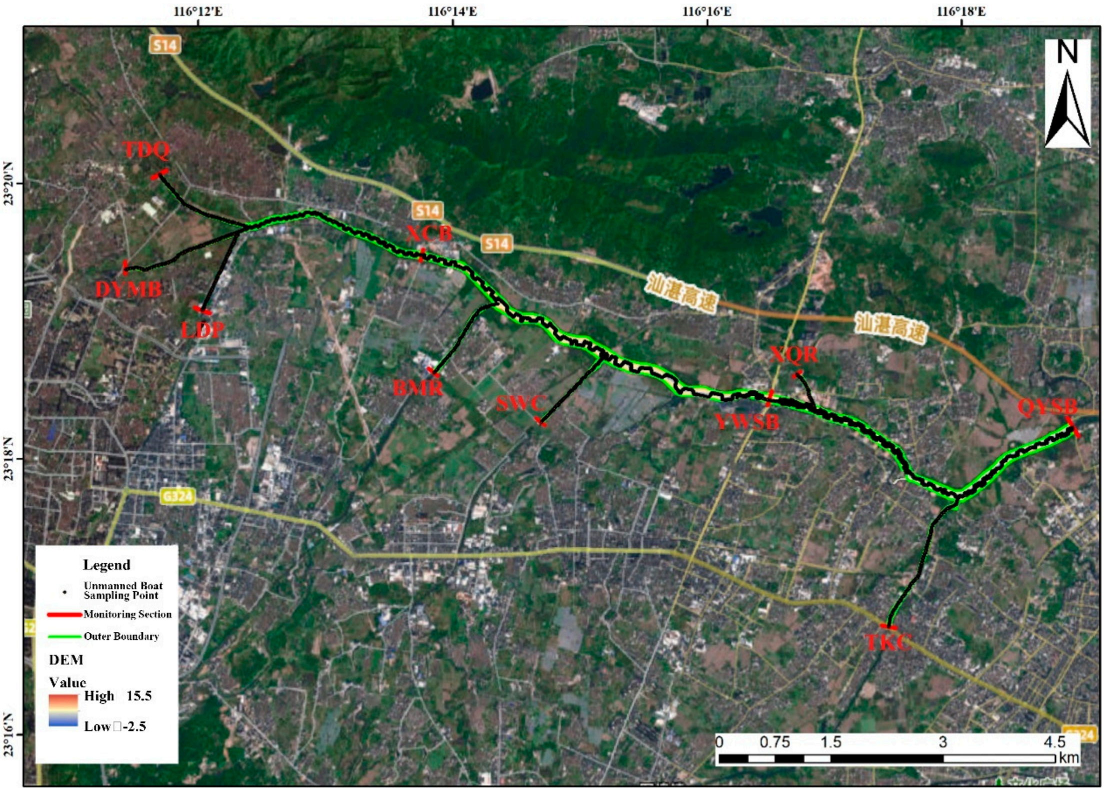

Fig. S1. Distribution of unmanned vessel bathymetry points and DEM in the Lianjiang River basin

3. Grid construction

In this study, the terrain mesh for the study area was generated using the SMS tool. The grid size for the main river inflow and outflow boundaries was set to 15 m, while the grid size for the tributary inflow boundaries ranged from 3 m to 5 m. The grid size for the external boundary of the main river was set to 18 m, and for the tributaries, the external boundary grid size ranged from 5 m to 10 m. Irregular triangular meshes were used, and after quality checks, a total of 18,485 grids were generated (Fig. S2).

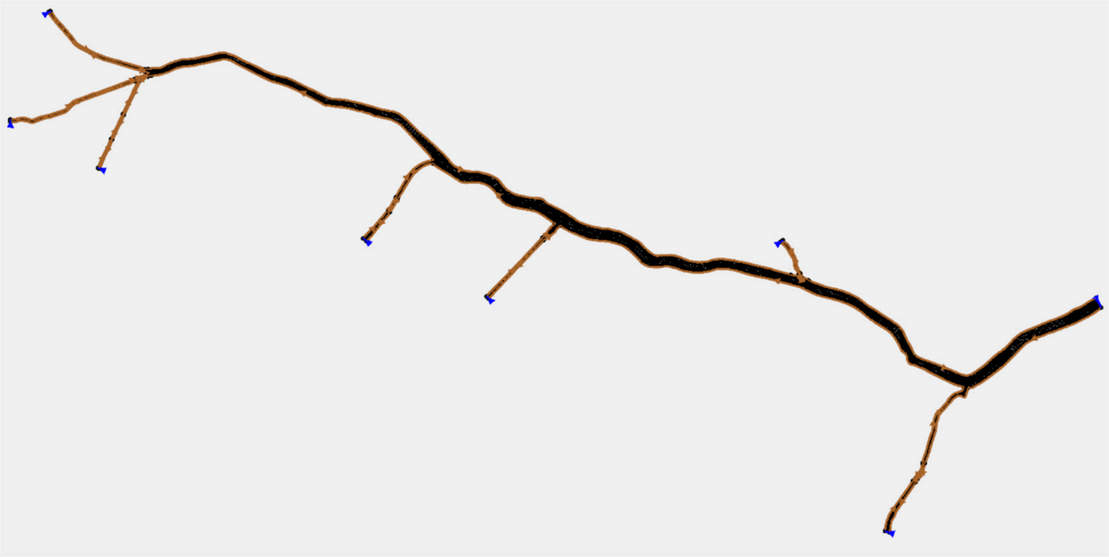

Fig. S2. Topographic grid map of the Lianjiang River basin

4. Terrain interpolation

In addition to the quality of the generated terrain mesh, the accuracy of the bathymetry is also critical for hydrodynamic model simulations. The terrain data in this study were divided into two parts: bathymetric data within the river channel were obtained from single-beam unmanned vessel bathymetry points, while water boundary and dam control point data were sourced from mobile RTK survey points. Given that the boundary of the study area consists of relatively simple riverbanks, the spline interpolation method was used to generate DEM data with a 1 m resolution for the study area (Fig. S1). Elevation data corresponding to the mesh node coordinates were then extracted from the generated triangular mesh, resulting in the final terrain mesh (Fig. S3).

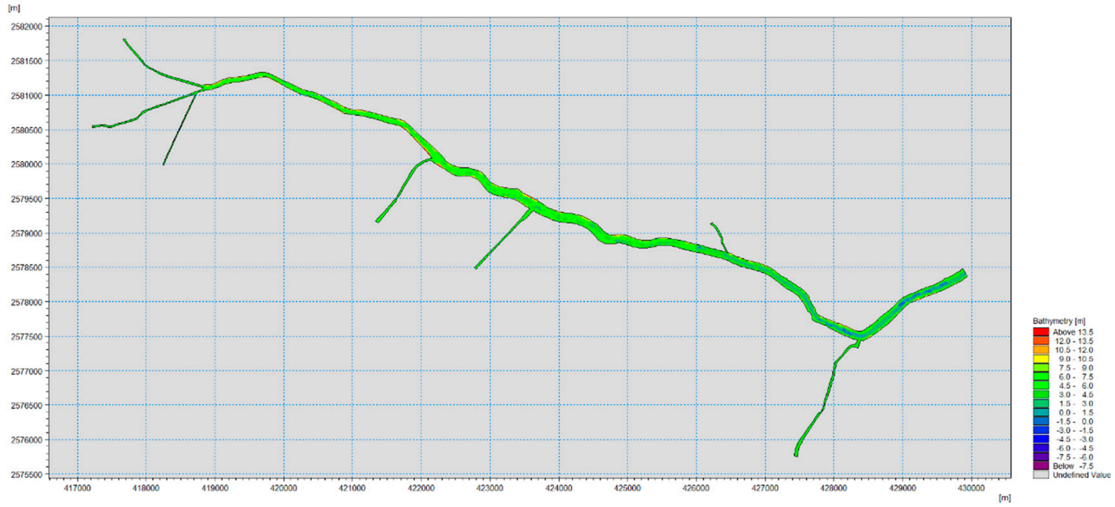

Fig. S3. Grid topographic map of the Lianjiang River Basin

5. Model parameter settings

5.1 Hydrodynamic module parameter settings

Closed boundary conditions: For all land boundaries, the flow variables perpendicular to the boundary are set to zero (no flow penetration). This implies that, in the momentum equation, the flow is completely stationary as the fluid approaches the land boundary.

Open boundary conditions: Open boundary conditions (in this study, for the upstream and downstream sections) can be defined as flow or water level control. Following common practice, to ensure rapid convergence and stability of the model, flow control is applied at the upstream boundary, using hourly flow data from seven hydrological monitoring stations (XCB, BMR, SWC, XQR, NQR, HZR, and TKC) in 2022 as input. At the downstream boundary, water level control is applied, with hourly discharge data from the QYSB station in 2022 used as the downstream boundary condition.

Dry-wet boundary conditions: To avoid computational instability, particularly when the model domain includes river channels and surrounding embankments, the "Flood and Dry" boundary option was enabled in this study. The dry water depth (h_{dry}), flood water depth (h_{flood}), and wet water depth (h_{wet}) were set to default values, satisfying the condition $h_{dry} < h_{flood} < h_{wet}$. The specific values were set as follows: $h_{dry} = 0.005$ m, $h_{flood} = 0.05$ m, and $h_{wet} = 0.1$ m.

CFL number: To ensure model stability, the CFL number must be less than 1. After multiple calibrations, the CFL number was ultimately set to 0.8 in this study.

Turbulent viscosity coefficient: To account for time- and space-dependent uncertainties in the physical processes, an additional stress term was introduced into the governing equations. The turbulent viscosity coefficient is used to characterize these processes by multiplying it with the average velocity gradient, which contributes to the effective shear stress in the momentum equation (including both laminar and Reynolds stresses). The turbulent viscosity coefficient in this study was set using the Smagorinsky formulation with a default value of 0.28.

Bottom friction: Bottom friction is represented by the Manning's coefficient. Given the length of the river channel in the Puning reach of the Lianjiang River and the varying resistance along the channel, a single value for resistance was deemed inappropriate. This study used a MATLAB-based roughness field generation program to calculate varying roughness values based on water depth, generating a spatially varying bottom friction file (.dfsu) (Fig. S4).

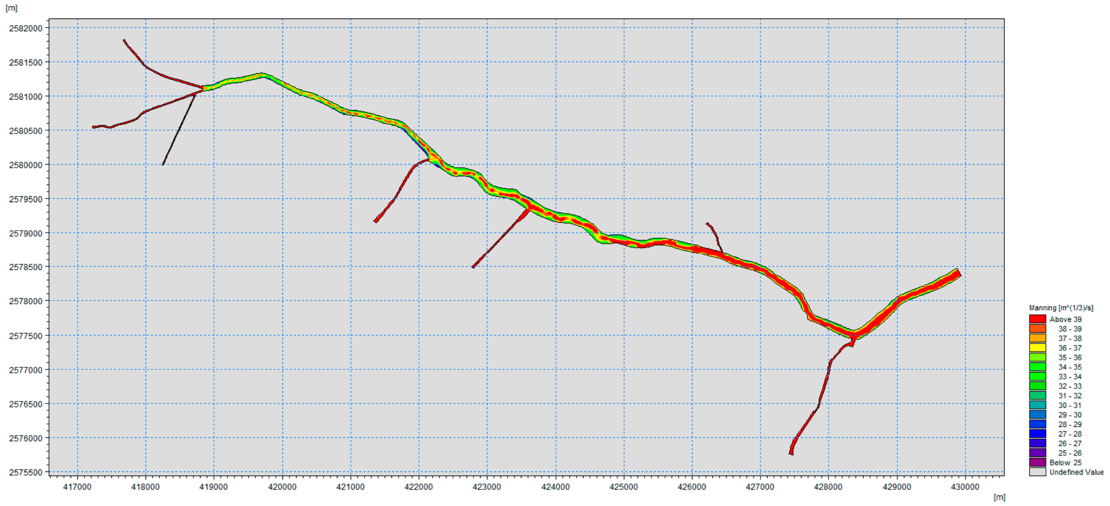

Fig. S4. Spatial variation of bottom friction in the Lianjiang River basin

Initial water level: Due to the long length of the study reach and significant water level variations, using a single water level value as the initial condition for the hydrodynamic module was not appropriate. Therefore, this study employed a warm start, using measured water levels at various locations within the river channel as the initial condition. A spatially varying water level file (.dfsu) was created to account for these spatial differences. This approach (Fig. S5) significantly improved the model's simulation accuracy.

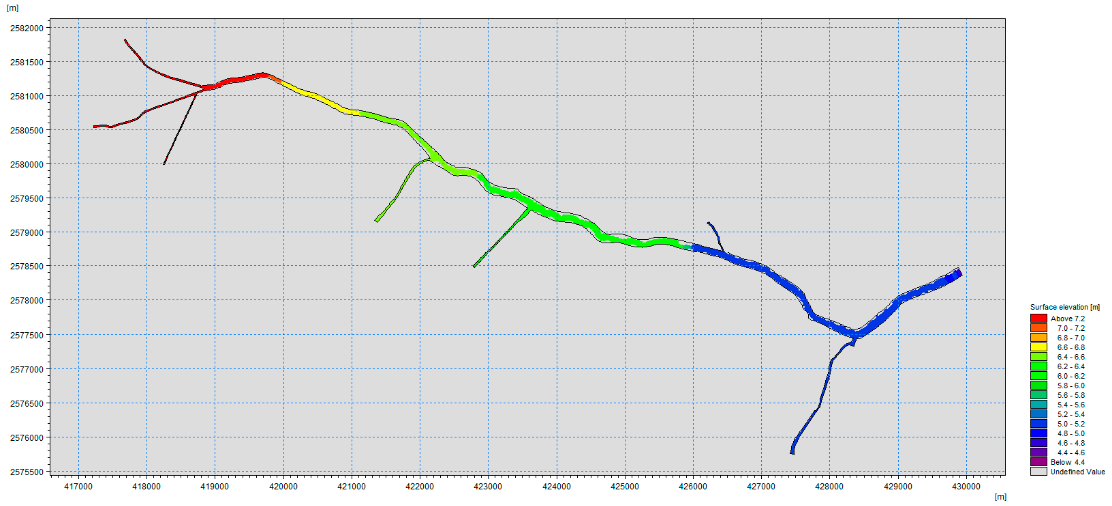

Fig. S5. Spatial variation of initial water levels for the hydrodynamic module

6. Water quality module parameter settings

Model analysis control factors: Based on the monitoring data from nine stations, it was found that DO, COD, NH_3-N, and TP are key water quality indicators for the Puning reach of the Lianjiang River basin. In line with the requirements for water quality management in water function zones, DO, COD, NH_3-N, and TP were selected as the control factors for model analysis.

Water quality boundary conditions: The required data for the water quality boundary conditions in the advection-dispersion module are pollutant concentrations at the upstream and downstream boundaries of the river. Water quality monitoring data from the XCB monitoring station for July and August 2022 were used as the upstream boundary conditions, while data from the QYSB station for the same months served as the downstream boundary conditions.

Initial water quality concentration settings: Due to the long length of the study reach and the presence of seven tributaries, water quality conditions vary significantly across different sections of the river. Therefore, it is not suitable to use a single water quality concentration for the entire river reach as the initial condition for the water quality module. Instead, a warm start method was used, where the initial concentrations of pollutants were set based on measured water quality data within the river. A spatially varying water quality concentration file (.dfsu) was created to represent these conditions. The spatial distribution of the initial water quality concentrations used as input for the model is shown in Figs. S6 to S9.

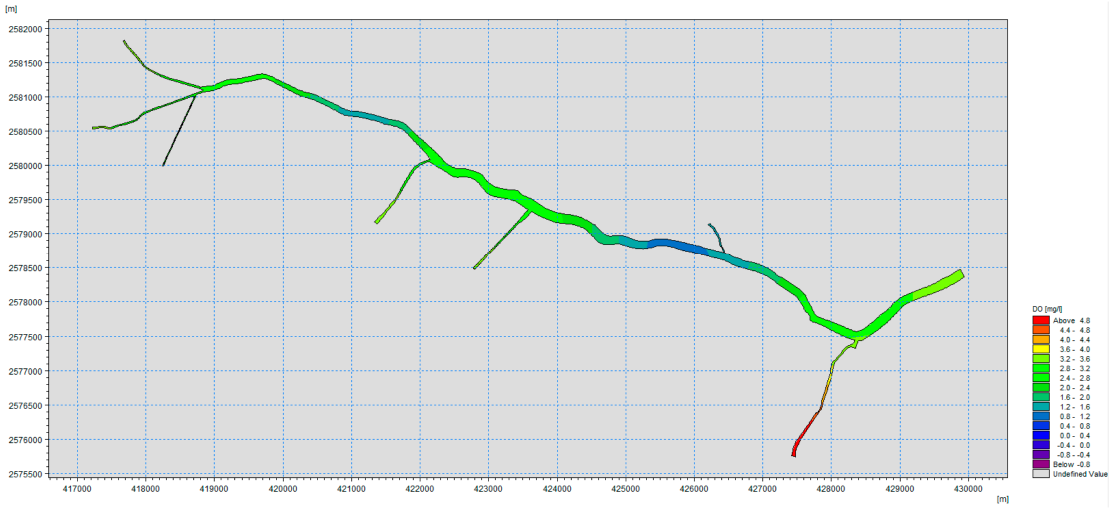

Fig. S6. Spatial variation of initial DO concentrations

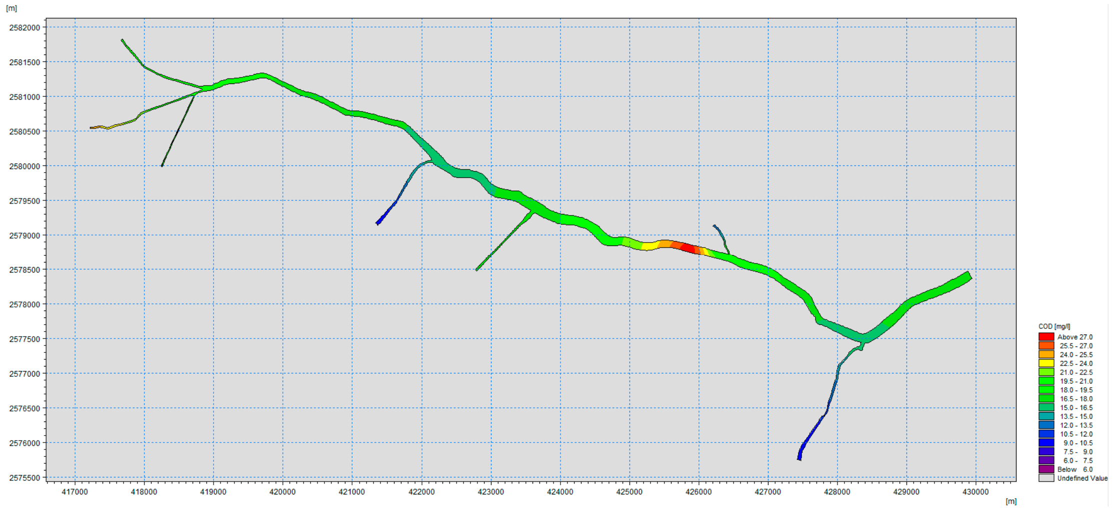

Fig. S7. Spatial variation of initial COD concentrations

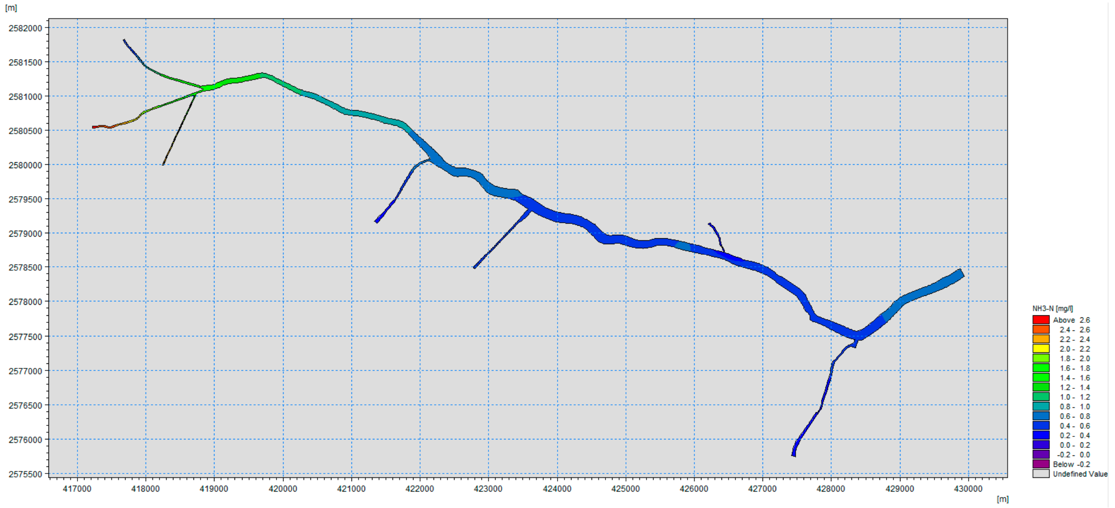

Fig. S8. Spatial variation of initial NH_3-N concentrations

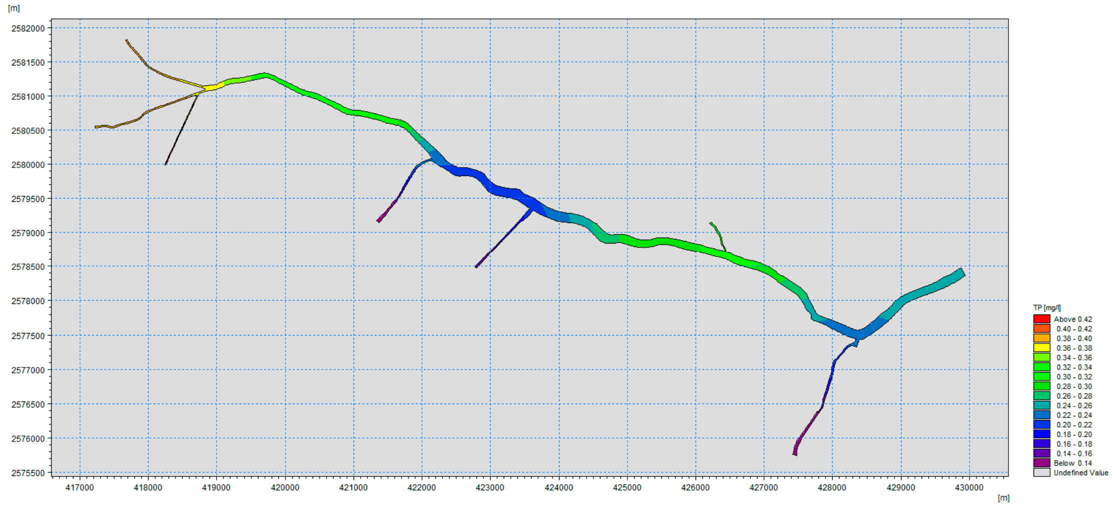

Fig. S9. Spatial variation of initial TP concentrations

Horizontal diffusion coefficient: The rate of pollutant transport and dispersion in the water is primarily determined by the diffusion coefficient, which is closely related to variations in the flow field. The typical range for horizontal diffusion coefficients is between 0.01 m^2/s and 20 m^2/s. Based on this range, and after multiple calibrations, model parameters for the Lianjiang River basin were finalized. The horizontal diffusion coefficients for DO, COD, NH_3-N, and TP were determined to be 0.2 m^2/s, 0.01 m^2/s, 0.1 m^2/s, and 0.5 m^2/s, respectively. These values will be further validated during the next Comprehensive pollutant degradation coefficient: The comprehensive pollutant degradation coefficient is a key parameter for evaluating the rate of pollutant attenuation in water bodies. The value of this coefficient is positively correlated with the rate of pollutant degradation. Pollutant degradation in water is a complex process influenced by various factors, encompassing multiple natural purification mechanisms: physical purification includes dilution, mixing, sedimentation, adsorption, and flocculation; chemical purification involves processes such as compound decomposition, acid-base neutralization, and redox reactions; and biological purification occurs through processes like biodegradation, biotransformation, and bioaccumulation. These purification processes typically occur simultaneously, but their duration and contribution to pollutant degradation vary. Therefore, to develop an accurate hydrodynamic-water quality coupled model, it is crucial to select and calibrate a comprehensive pollutant degradation coefficient that closely reflects the actual environmental conditions. After multiple calibrations, the degradation coefficients for DO, COD, NH_3-N, and TP in the Puning reach of the Lianjiang River basin were

determined to be -0.22 mg/d, 0.04 mg/d, 0.09 mg/d, and 0.10 mg/d, respectively. These values will be further confirmed during the next phase of model validation.

In summary, the main parameters for the hydrodynamic model simulation include the dry-wet boundary conditions, CFL number, turbulent viscosity coefficient, and bottom friction. The primary parameters for the water quality model simulation are the diffusion coefficient and pollutant degradation coefficients. The final parameter calibration results for the MIKE 21 model are presented in Table S1.

Table S1. Model parameter settings

Parameter	Value	Parameter	Value
Dry/wet boundary / (m)	h_{dry}=0.005<br>h_{flood}=0.05<br>h_{wet}=0.1	NH_3-Ndiffusion coefficient (m^2/s)	0.1
CFL number	0.8	TP diffusion coefficient (m^2/s)	0.5
Turbulent viscosity coefficient (m^2/s)	0.28	DO degradation coefficient (mg/d)	-0.22
Bottom friction ($m^{1/3}/s$)	spatial sequence values	COD degradation coefficient (mg/d)	0.04
DO diffusion coefficient (m^2/s)	0.2	NH_3-N degradation coefficient (mg/d)	0.09
COD diffusion coefficient (m^2/s)	0.01	TP degradation coefficient (mg/d)	0.10

The software version used in this study is DHI MIKE 21 2014 (MIKE Zero platform). The time step was set with a minimum of 0.01 s and a maximum of 300 s, and the output time step was 3600 s. The MIKE 21 HD module solves the shallow water equations using an explicit finite volume scheme, with time advancement controlled by the CFL condition for stability. In this study, computational stability was ensured by controlling the CFL number (0.8) and the range of time steps.

Text S2 Preliminary diagnosis of water pollution sources

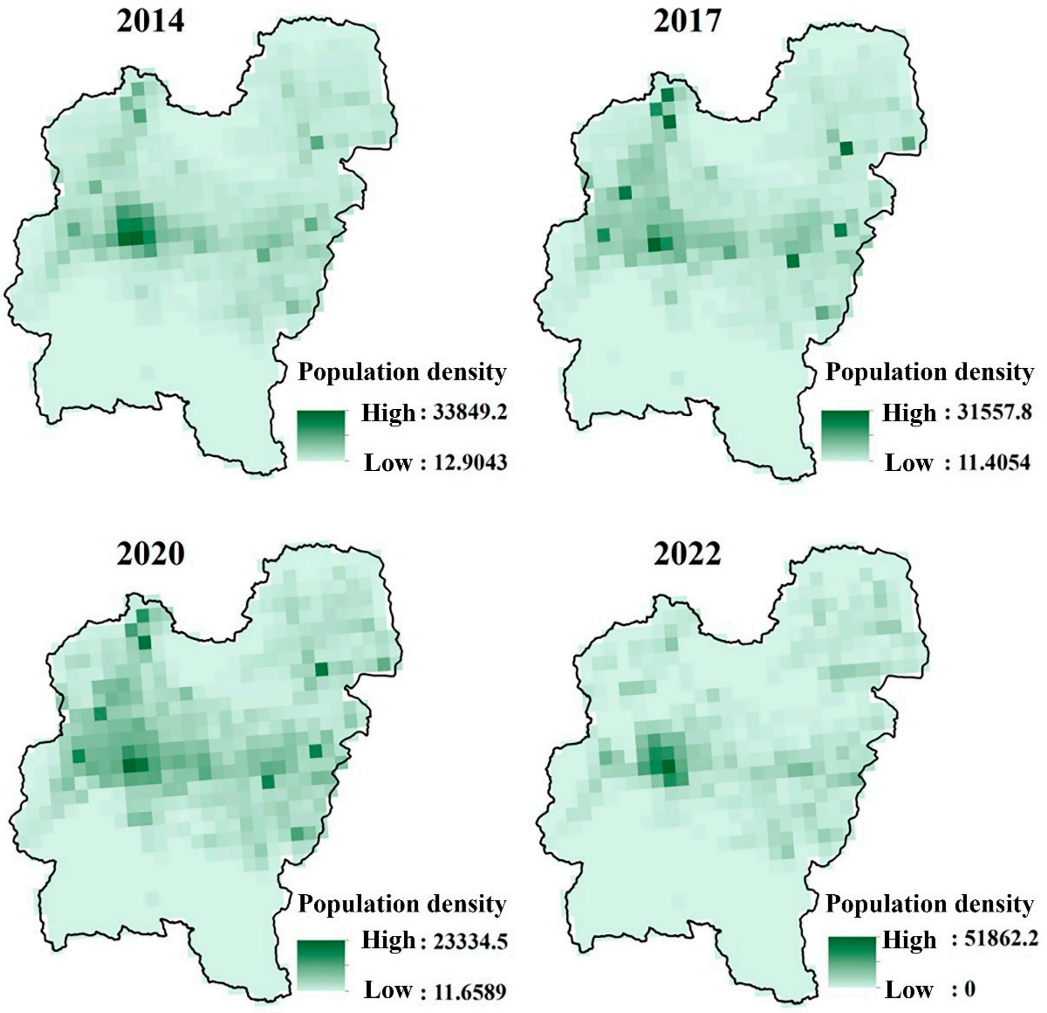

Fig. S10. Population density in the Puning reach of the Lianjiang River basin

Table S2. Puning city's annual resident population and national economic data from 2014 to 2020

Year	Year-end resident population (10^4)	Gross value of primary industry (10^4 yuan)	Gross value of secondary industry (10^4 yuan)	Gross value of tertiary industry (10^4 yuan)
2014	210.08	338063	3739540	1509580
2015	210.73	364950	3860892	1685739
2016	211.05	411929	4082672	1852078
2017	210.70	308361	4230448	2106677

2018				
2019	212.3	396580	2141586	3587978
2020	199.98	434399	2036970	3664472
2021	201.71	442385	1776333	3857132
2022	202.49	479095	1989634	3826263

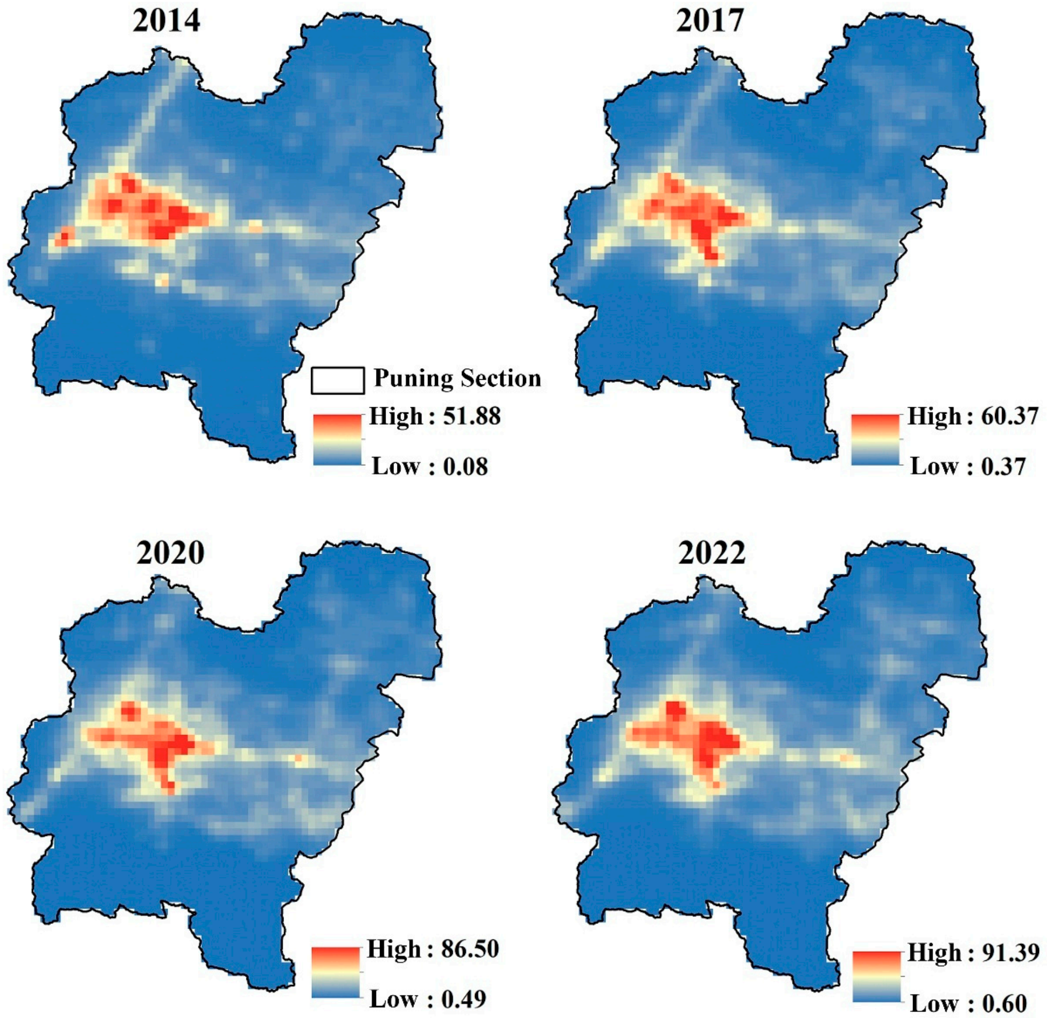

Fig. S11. Distribution of night-time lights in the Puning reach of the Lianjiang River basin from 2014 to 2022

Table S3. Number of industrial enterprises in Puning city from 2014 to 2022

Year	Light industry	Heavy industry	Total
2014	480	66	546
2015	513	66	579
2016	486	61	547

2017	470	59	529
2018			
2019	260	44	304
2020	248	52	300
2021	239	59	298
2022	276	71	347

Table S4. Number of livestock and poultry in Puning city from 2014 to 2022

Year	Rural population	Year-end cattle stock	Year-end pig stock (10^4)	Year-end sheep population	Year-end chicken stock (10^4)	Year-end duck stock (10^4)
2014	1843551	26560	28	720	97	94
2015	1868665	25153	25	673	96	93
2016	1913916	19850	21.85	1020	74.36	64.76
2017	1955981	16899	18	2860	99	32
2018	/	/	/	/	/	/
2019	1377611	13646	13.55	1450	46.32	38.28
2020	1390709	14151	11.20	1520	44.75	35
2021	1395527	13511	15.78	1493	53.22	28.58
2022	1397334	12427	21.60	1661	57.17	19.94

Table S5. Fertilizer application in Puning city from 2014 to 2022

Year	Fertilizer application for agriculture (t)	Nitrogen fertilizer (t)	Phosphorus fertilizer (t)	Potassium fertilizer (t)	Compound fertilizer (t)
2014	31044				
2015	31065				
2016	31072				
2017	26474				
2018					
2019	28332	17392	2520	5865	2555
2020	28377	17393	2538	5865	2581
2021	28095	17334	2517	5817	2427
2022	28068	17317	2515	5811	2425

Text S3 Effects of different inflow interception scenarios on water quality improvement in the Puning reach of the Lianjiang River basin

Table S6. Inflow conditions at the monitoring sections

Monitoring section	Discharge (m^3/s)	COD (mg/L)	DO (mg/L)	NH_3-N (mg/L)	TP (mg/L)
TDB	3.49	24.20	2.71	2.96	0.20
DYMB	1.94	22.18	2.53	1.48	0.25
LDP	0.82	22.80	1.78	3.09	0.61
BMR	1.20	12.58	5.68	0.13	0.04
SWC	0.42	13.10	4.68	0.29	0.09
XQR	0.18	21.32	4.10	1.28	0.13
TKC	0.83	9.79	5.50	0.13	0.08

Table S7. Initial water quality conditions at the monitoring sections before interception (mg L^{-1})

Monitoring section	COD	DO	NH_3-N	TP
XCB	23.25	2.69	2.49	0.27
YWSB	21.00	3.63	1.95	0.22
QYSB	19.28	4.77	1.72	0.19

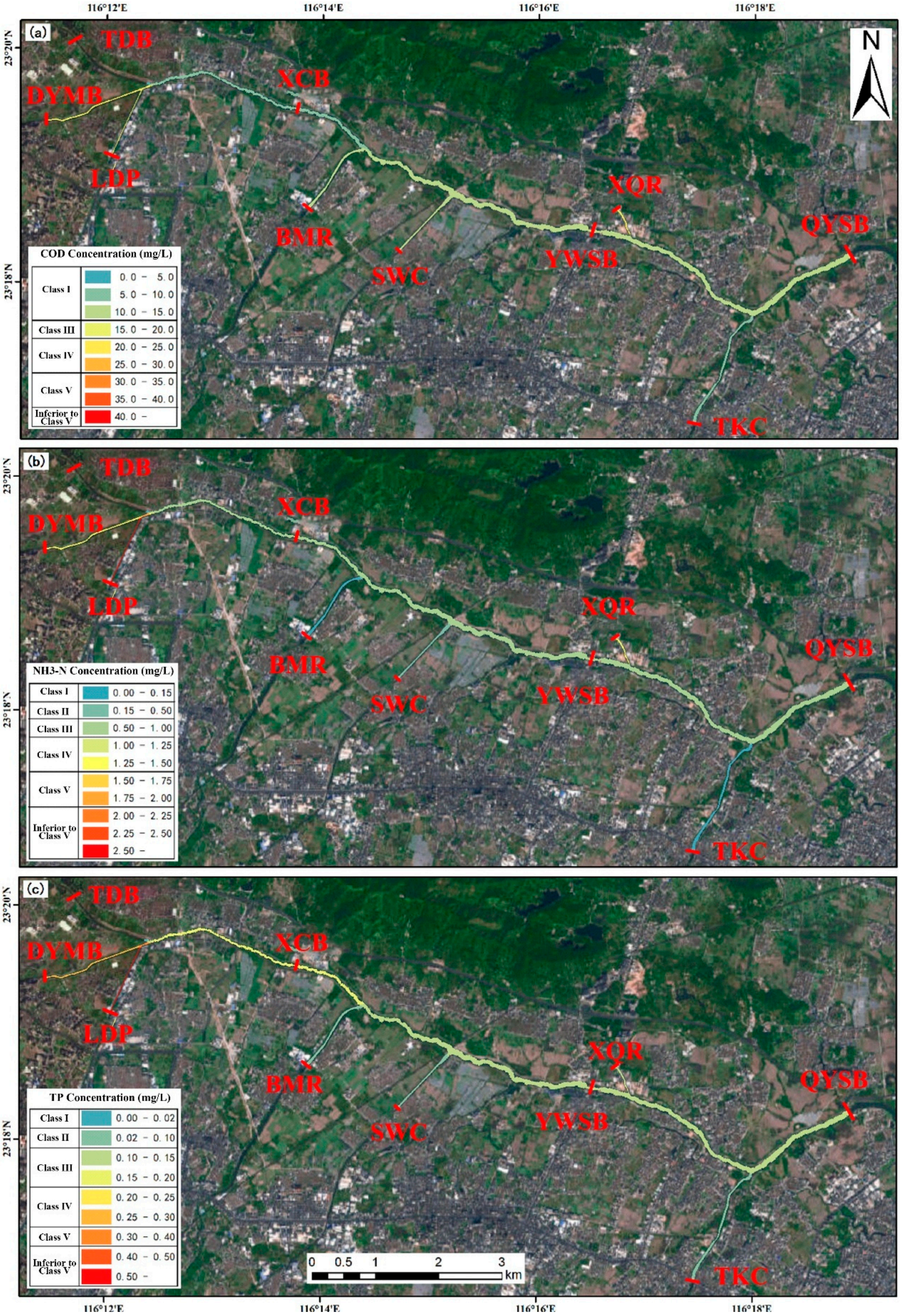

Fig. S12. Spatial distribution of pollutant loads under Scenario 1 (interception at the TDB section on the BKLR); (a) COD; (b) NH_3-N; (c) TP

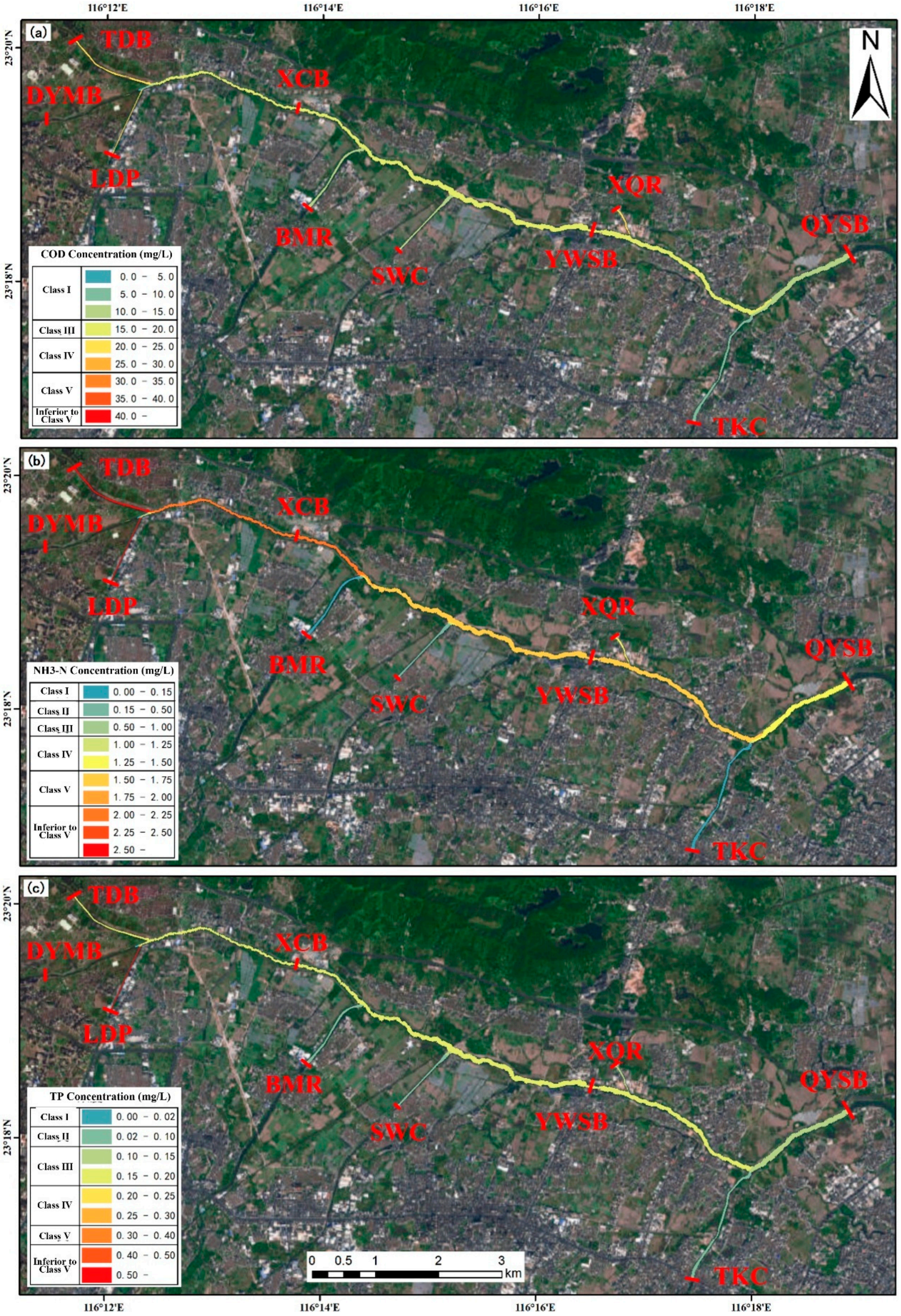

Fig. S13. Spatial distribution of pollutant loads under Scenario 2 (interception at the DYMB section on the LSXR); (a) COD; (b) NH_3-N; (c) TP

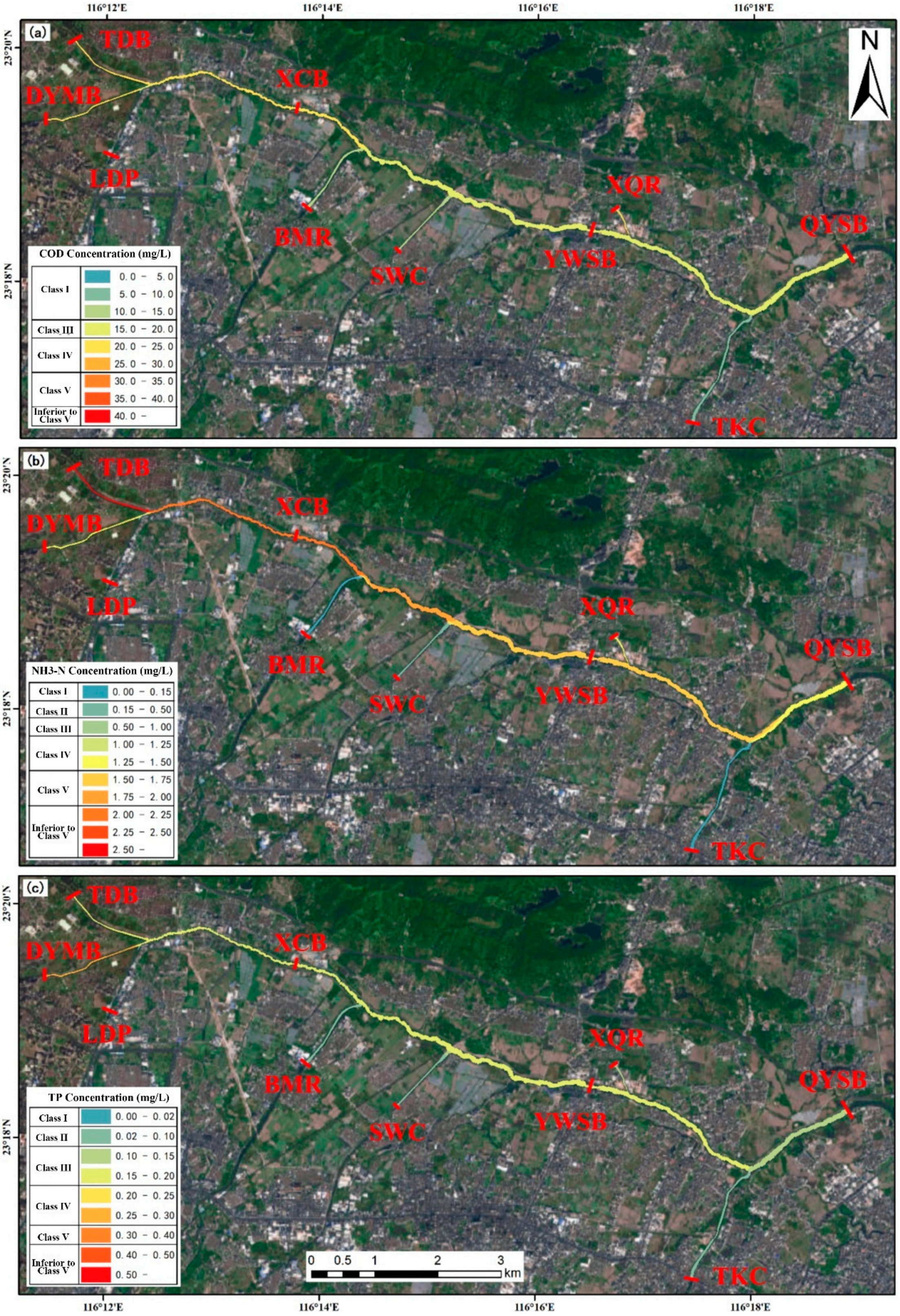

Fig. S14. Spatial distribution of pollutant loads under Scenario 3 (interception at the LDP section on the LSZR); (a) COD; (b) NH_3-N; (c) TP

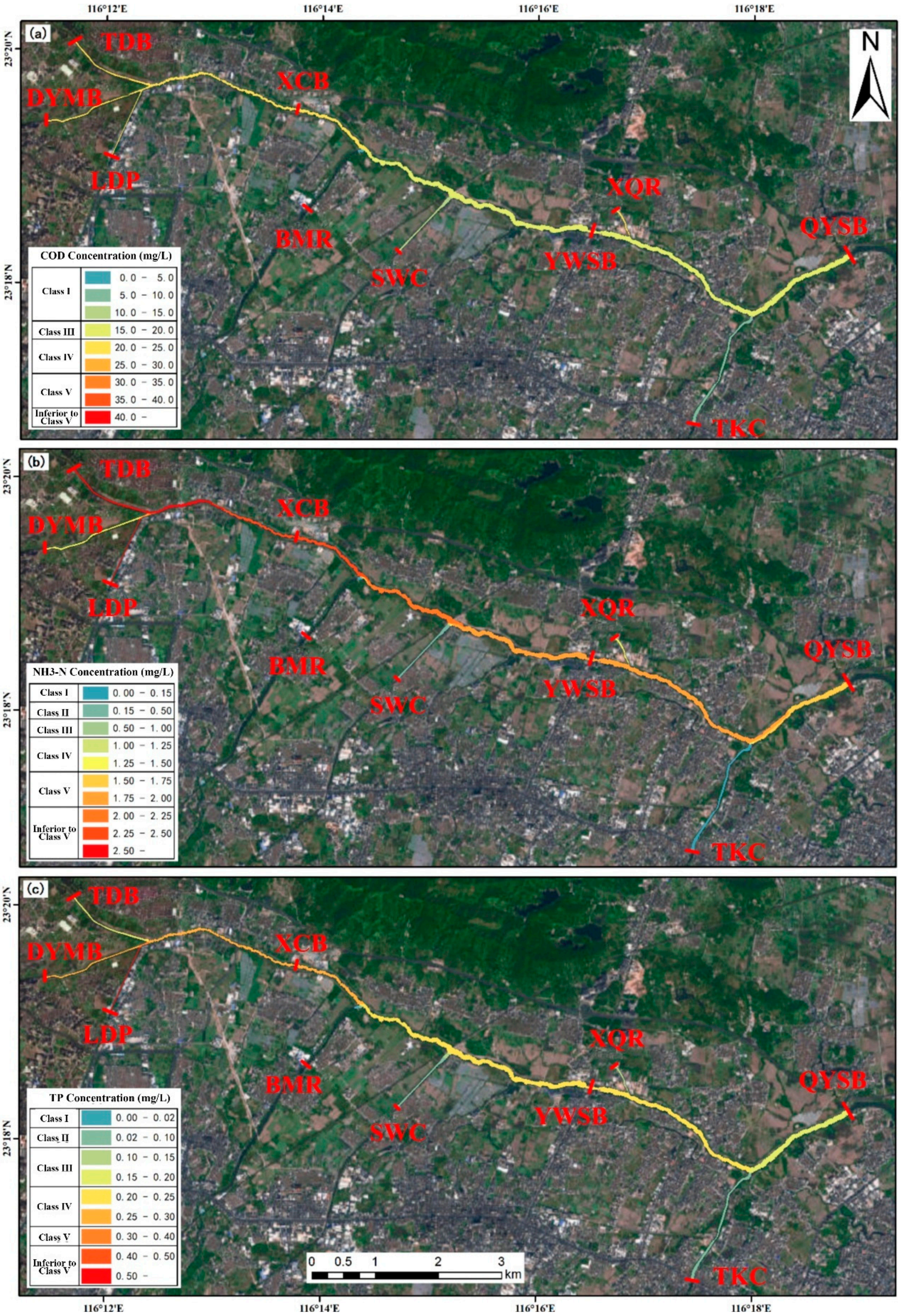

Fig. S15. Spatial distribution of pollutant loads under Scenario 4 (interception at the BMR section); (a) COD; (b) NH_3-N; (c) TP

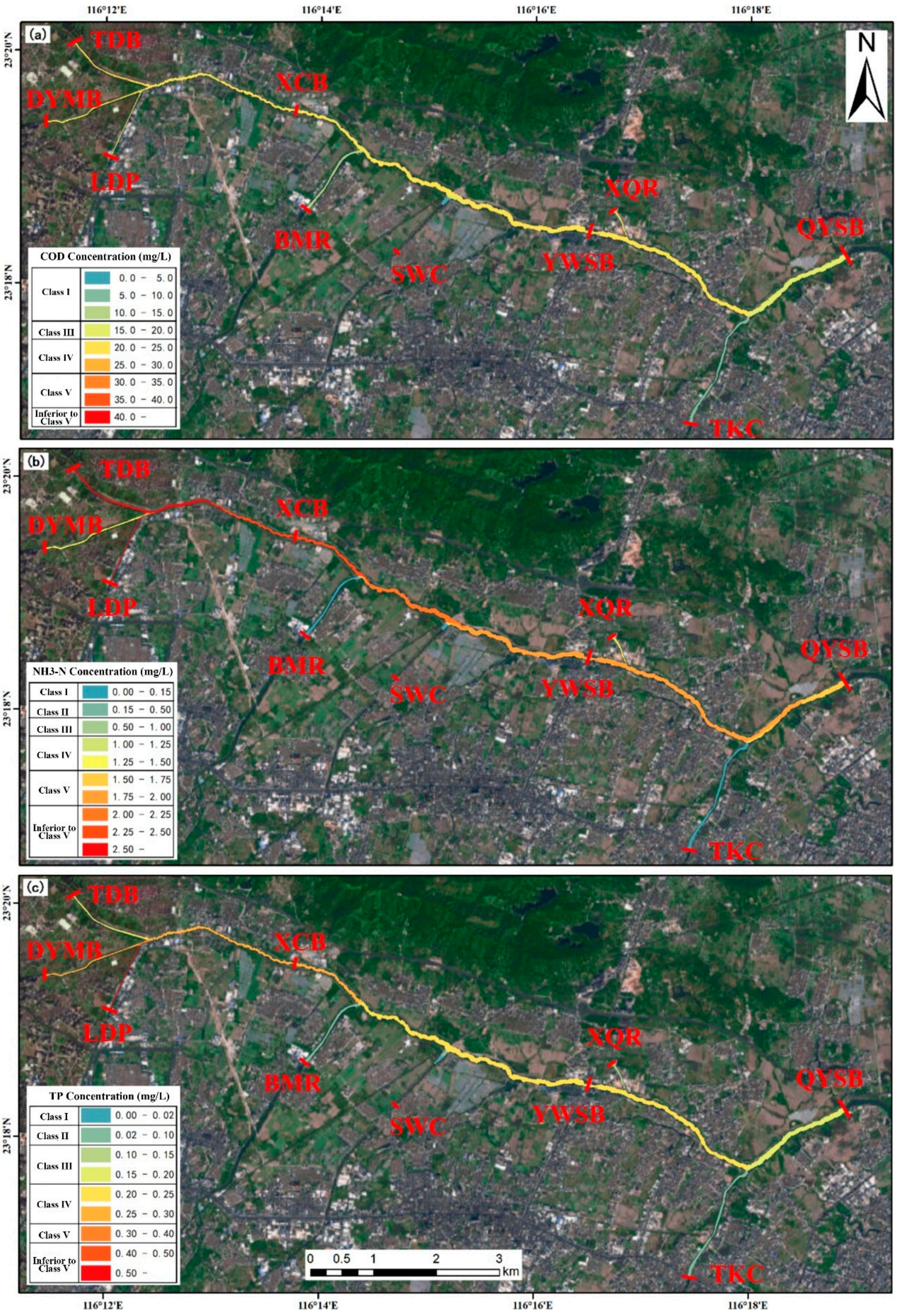

Fig. S16. Spatial distribution of pollutant loads under Scenario 5 (interception at the SWC section); (a) COD; (b) NH_3-N; (c) TP

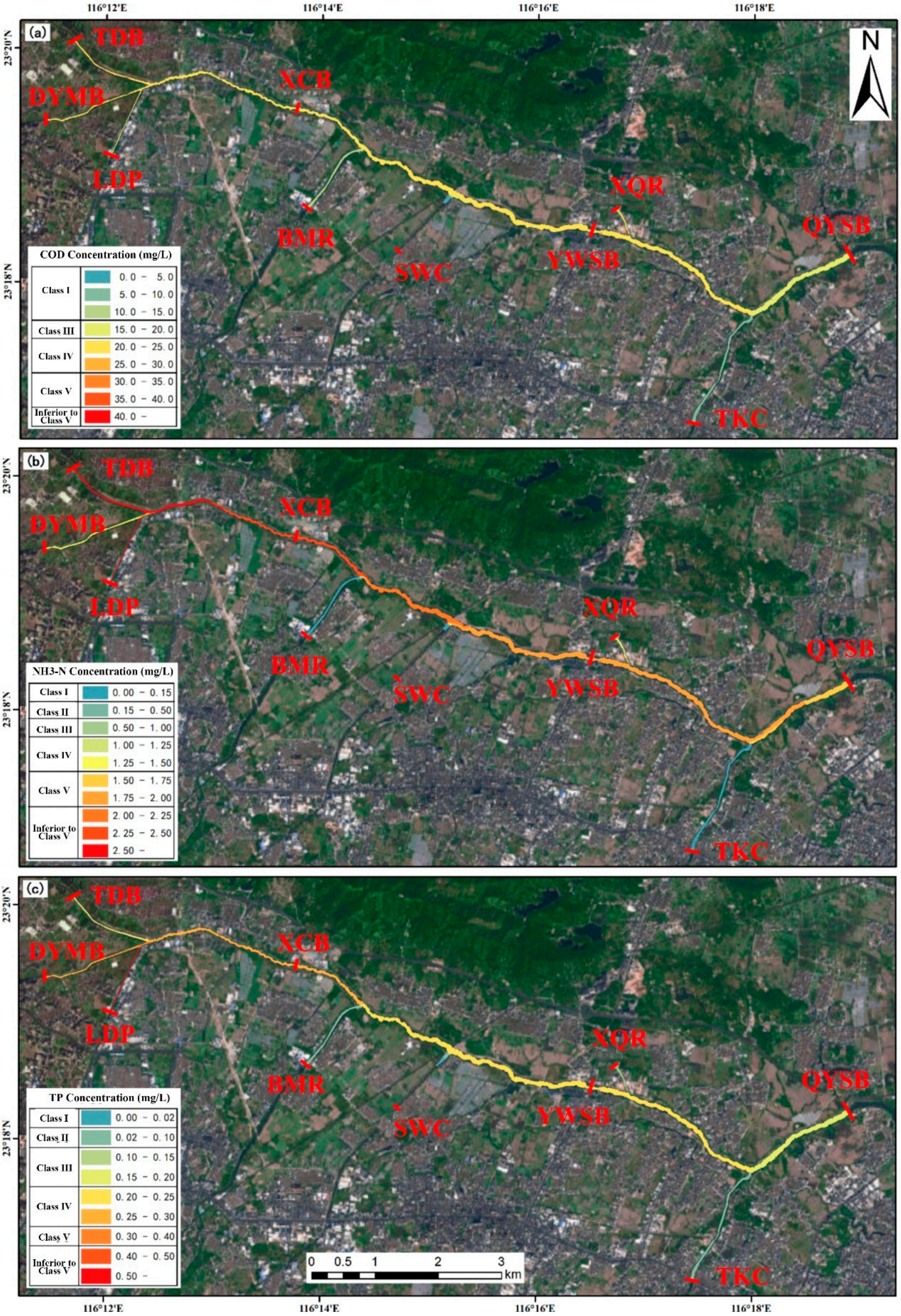

Fig. S17 Spatial distribution of pollutant loads under Scenario 6 (interception at the XQR section); (a) COD; (b) NH_3-N; (c) TP

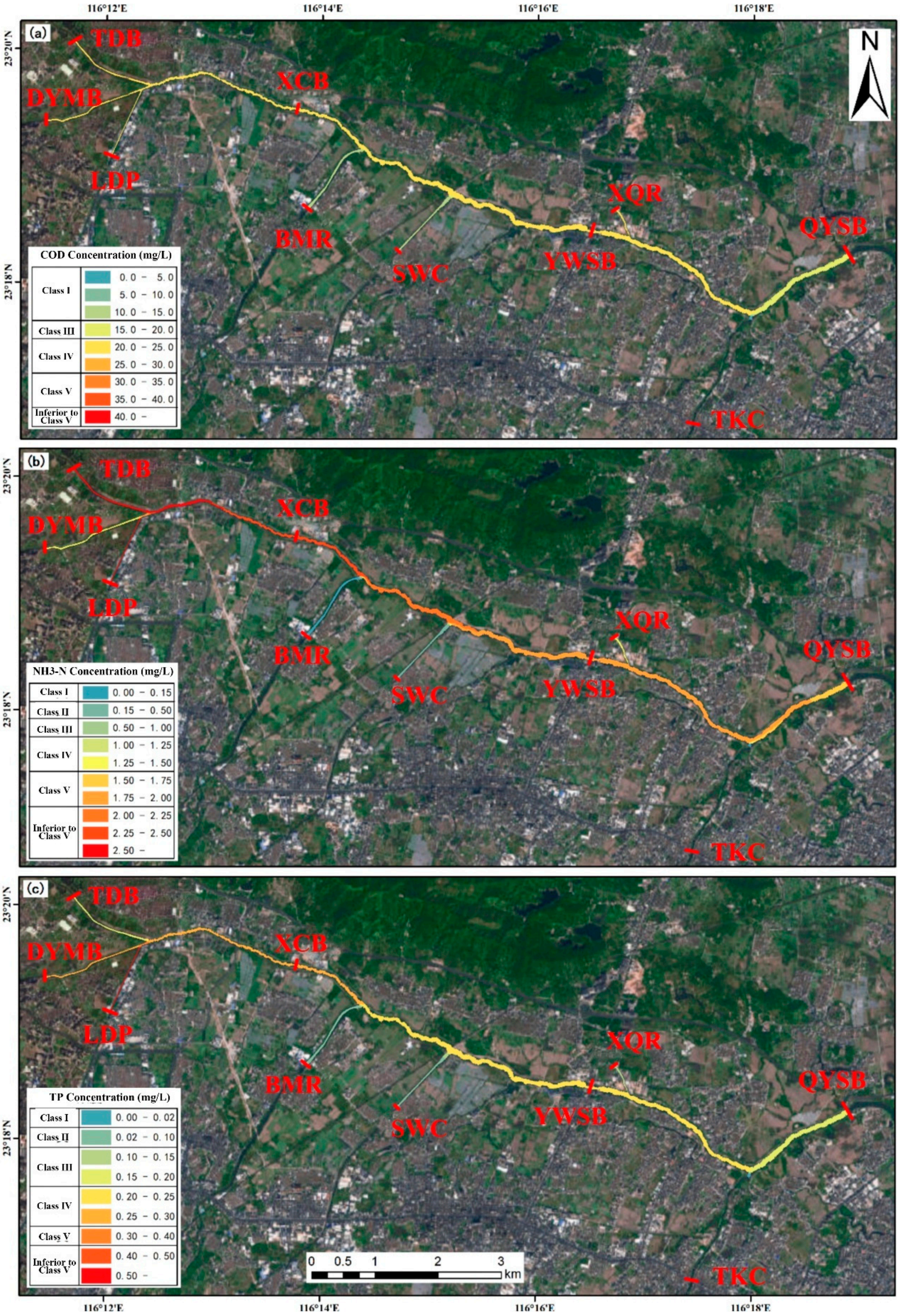

Fig.S18. Spatial distribution of pollutant loads under Scenario 7 (interception at the TKC section); (a) COD; (b) NH_3-N; (c) TP

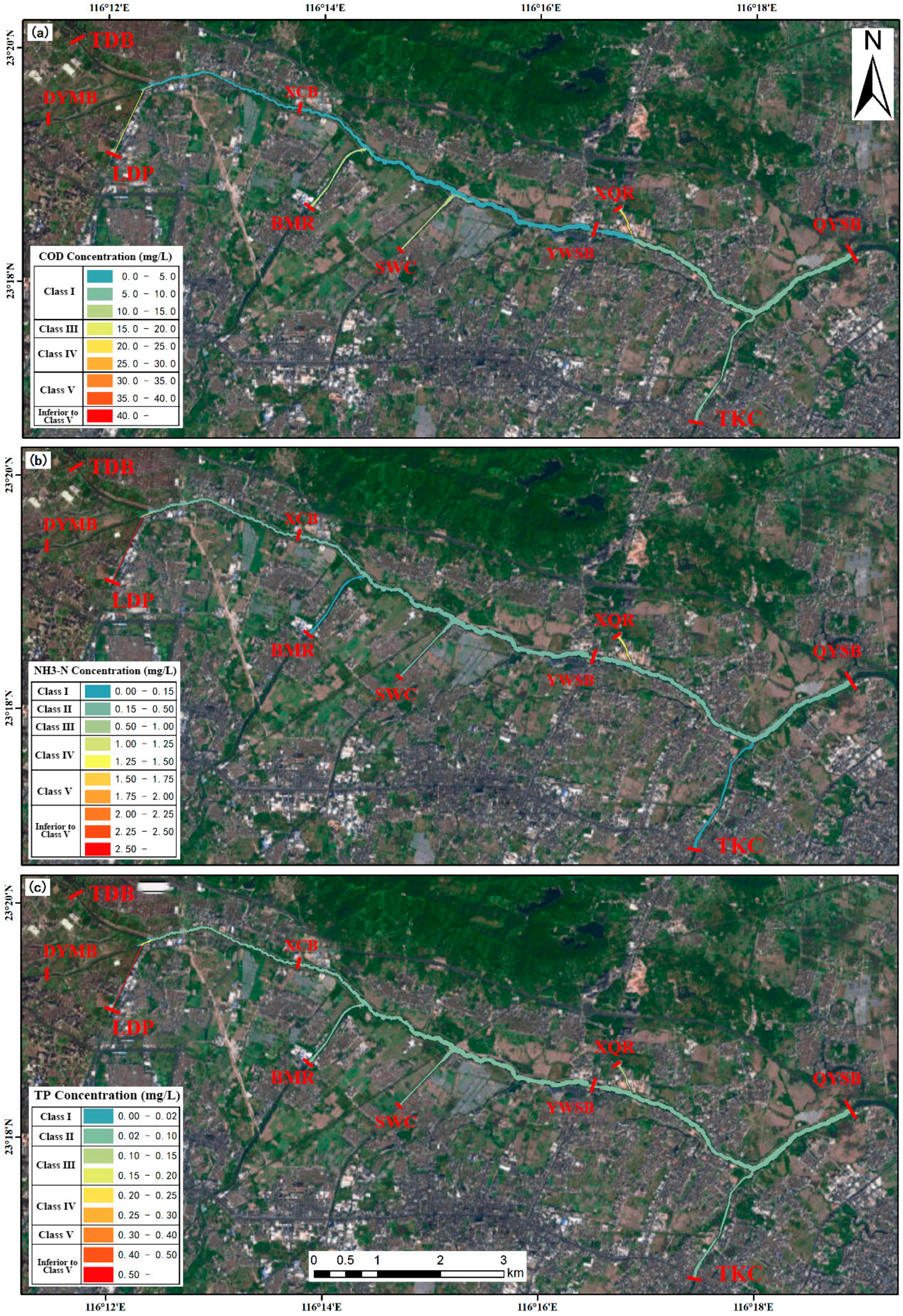

Fig.S19. Spatial distribution of pollutant loads under Scheme 8 (simultaneous interception at the TDB section of BKLR and the DYMB section of the LSXR River): (a) COD; (b) NH_3–N; (c) TP

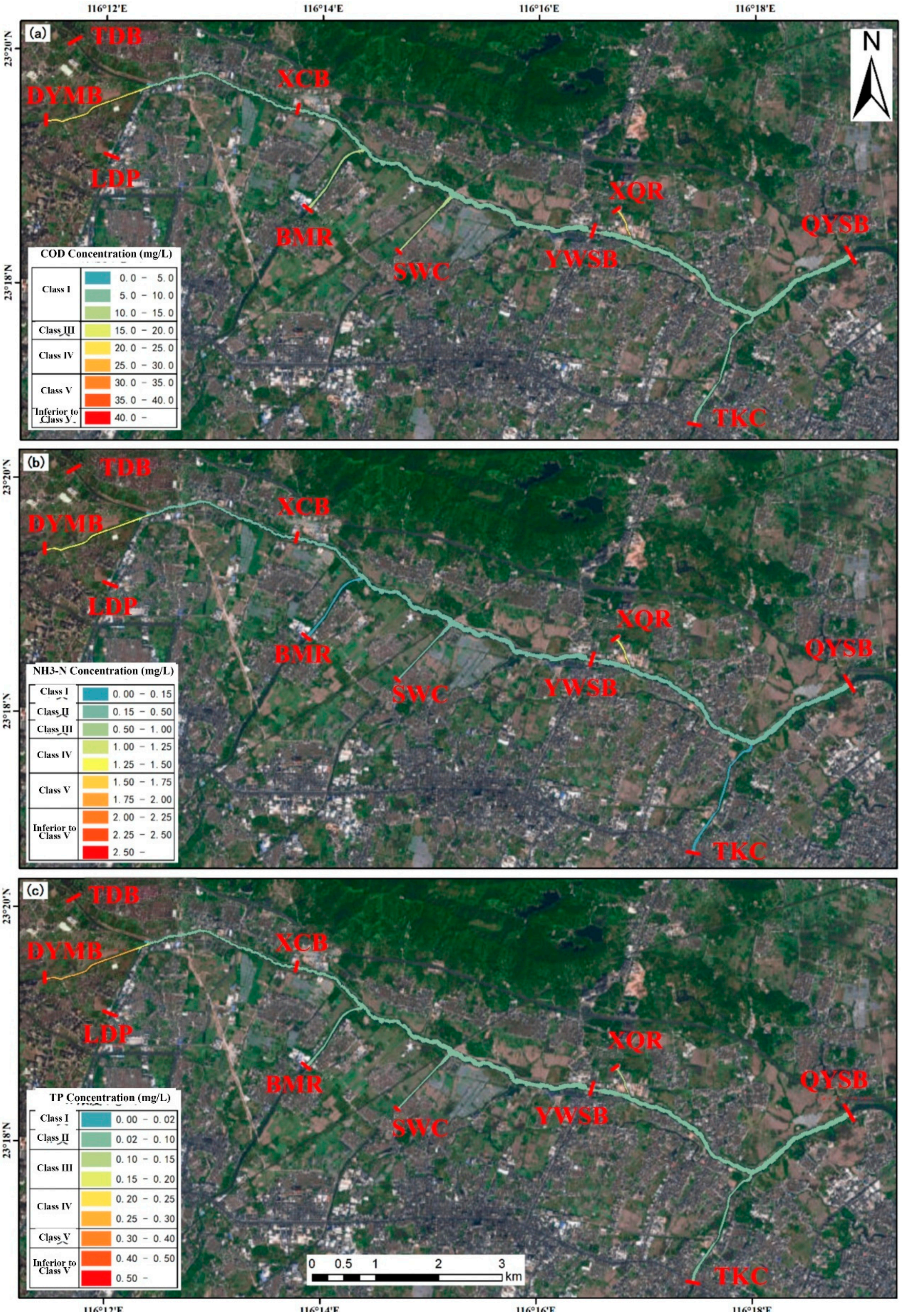

Fig. S20. Spatial distribution of pollutant loads under Scenario 9 (simultaneous interception at TDB on the BKLR and LDP on the LSZR): (a) COD; (b) NH_3-N; (c) TP

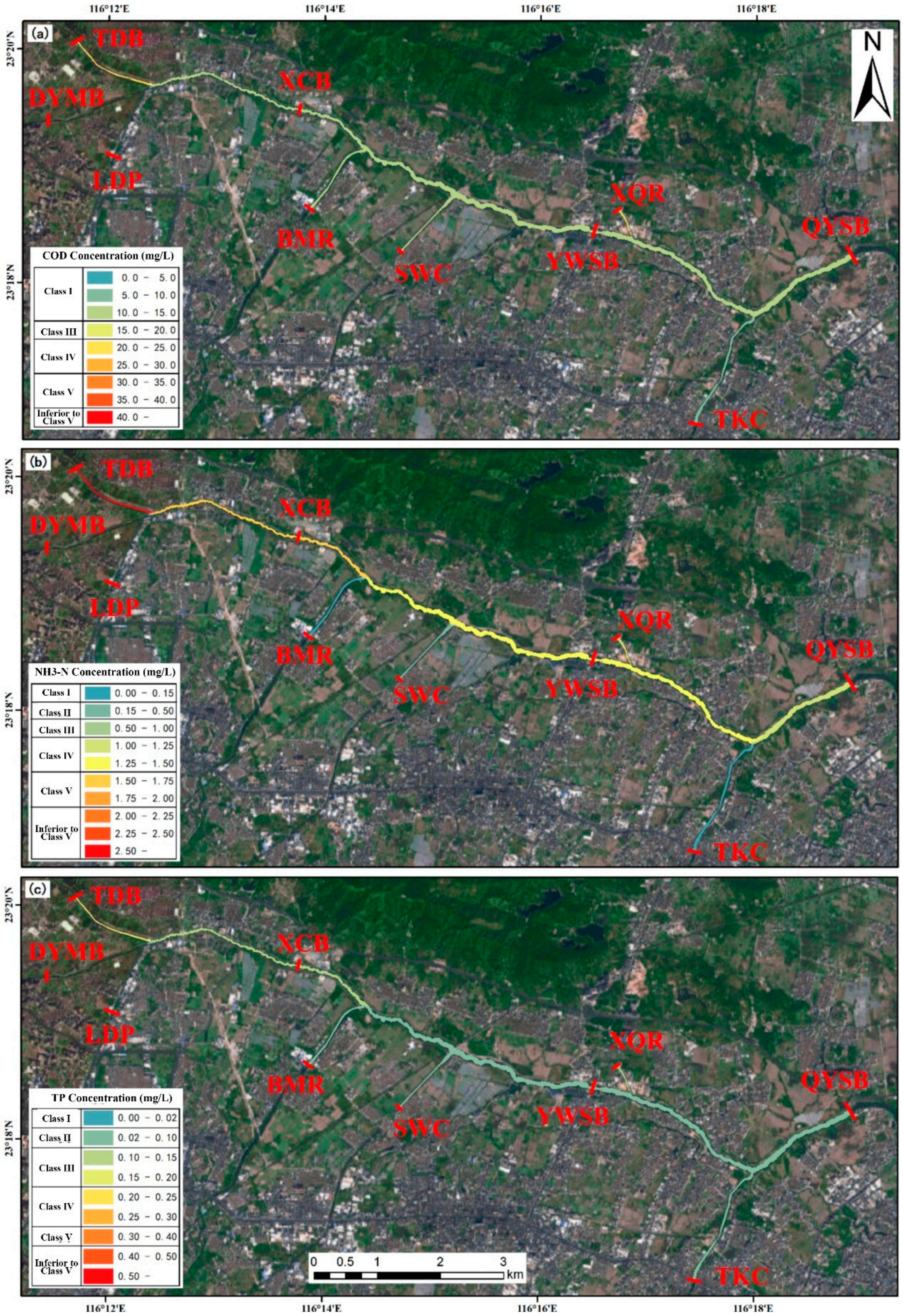

Fig. S21. Spatial distribution of pollutant loads under Scenario 10 (simultaneous interception at DYMB on the LSXR and LDP on the LSZR): (a) COD; (b) NH_3-N; (c) TP

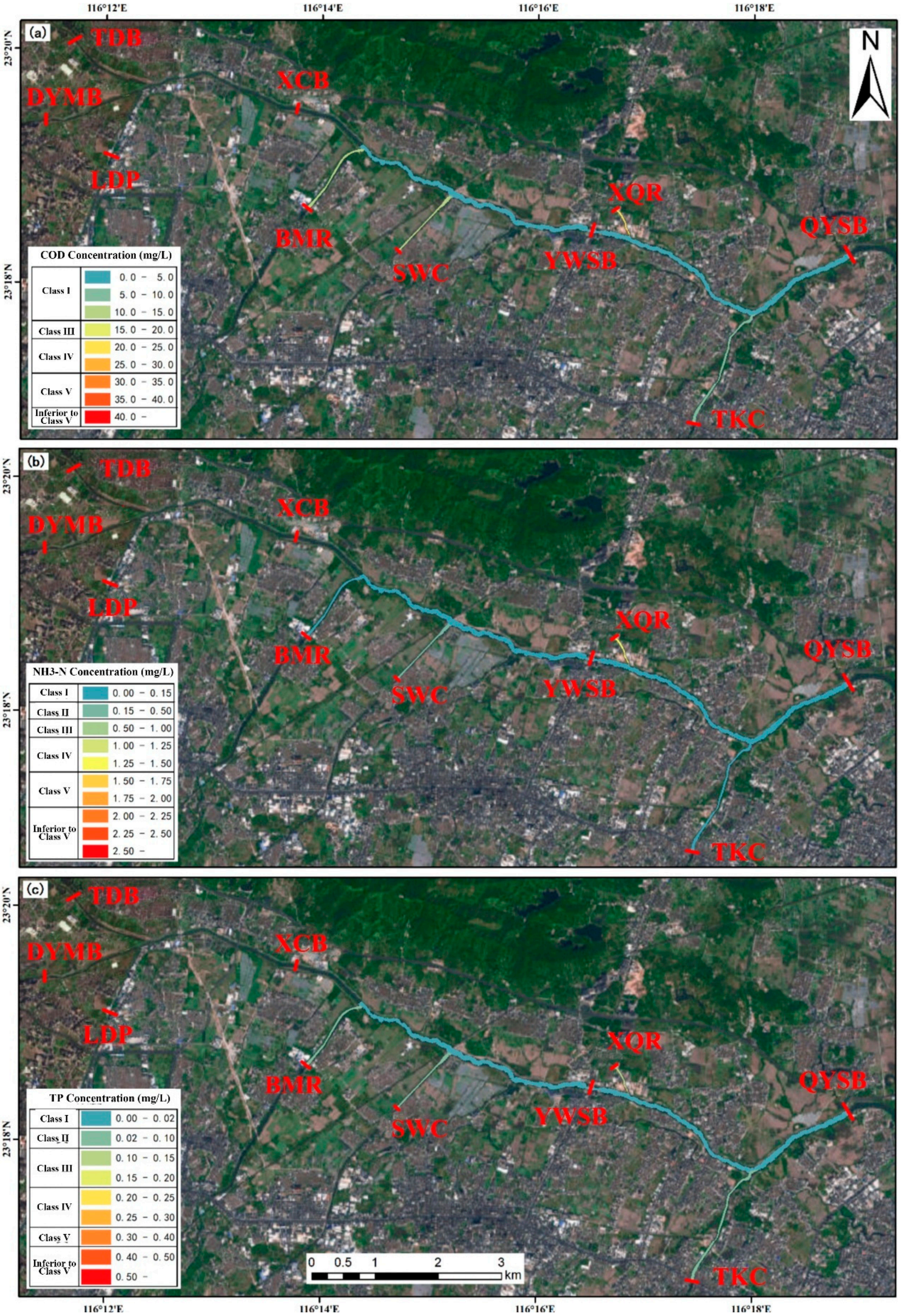

Fig. S22. Spatial distribution of pollutant loads under Scenario 11 (simultaneous interception at TDB on the BKLR, DYMB on the LSXR, and LDP on the LSZR): (a) COD; (b) NH_3-N; (c) TP

Text S4 Water quality improvements under different key engineering intervention scenarios in the Puning reach of the Lianjiang River basin

The Puning Municipal Wastewater Purification Plant is located downstream of the DYMB; the Huaxi Village integrated drainage-channel treatment facility is situated downstream of the LDP sluice gate; and the newly proposed treatment facility in the scenario setting is located downstream of the TDB. All three treatment facilities are positioned upstream of the confluences where their respective tributaries discharge into the main stream. Specifically, the Huaxi Village integrated drainage-channel treatment facility has an average daily inflow of 2,400 tons, with average daily effluent concentrations of 14.46 mg/L for COD, 0.10 mg/L for ammonia nitrogen, and 0.23 mg/L for total phosphorus. The Puning Municipal Wastewater Purification Plant has an average daily inflow of 40,000 tons, with average daily effluent concentrations of 5.58 mg/L for COD, 0.07 mg/L for ammonia nitrogen, and 0.15 mg/L for total phosphorus. The simulated new wastewater treatment facility downstream of the TDB is designed with reference to the Puning Plant, with an average daily inflow of 40,000 tons and average daily effluent concentrations of 14.70 mg/L for COD, 2.15 mg/L for ammonia nitrogen, and 0.14 mg/L for total phosphorus.

Table S8. Scenario settings for four key engineering interventions (mg/L)

Scenarios	Pollutants	TDB	DYMB	LDP	BMR	SWC	XQR	TKC
Discharge(m^3/s)		3.49	1.94	0.82	1.20	0.42	0.18	0.83
Scenario 1	COD	24.20	5.58	14.46	12.58	13.10	21.32	9.79
	NH_3-N	2.96	0.07	0.10	0.13	0.29	1.28	0.13
	TP	0.20	0.15	0.23	0.04	0.09	0.13	0.08
Scenario 2	COD	24.20	5.58	22.80	12.58	13.10	21.32	9.79
	NH_3-N	2.96	0.07	3.09	0.13	0.29	1.28	0.13
	TP	0.20	0.15	0.61	0.04	0.09	0.13	0.08
Scenario 3	COD	24.20	22.18	14.46	12.58	13.1	21.32	9.79
	NH_3-N	2.96	1.48	0.10	0.13	0.29	1.28	0.13

	TP	0.20	0.25	0.23	0.04	0.09	0.13	0.08
Scenario 4	COD	14.70	5.58	14.46	12.58	13.10	21.32	9.79
	NH_3-N	2.15	0.07	0.10	0.13	0.29	1.28	0.13
	TP	0.14	0.15	0.23	0.04	0.09	0.13	0.08

Text S5 Water quality improvements under integrated water environment management scenarios in the Puning reach of Lianjiang River basin

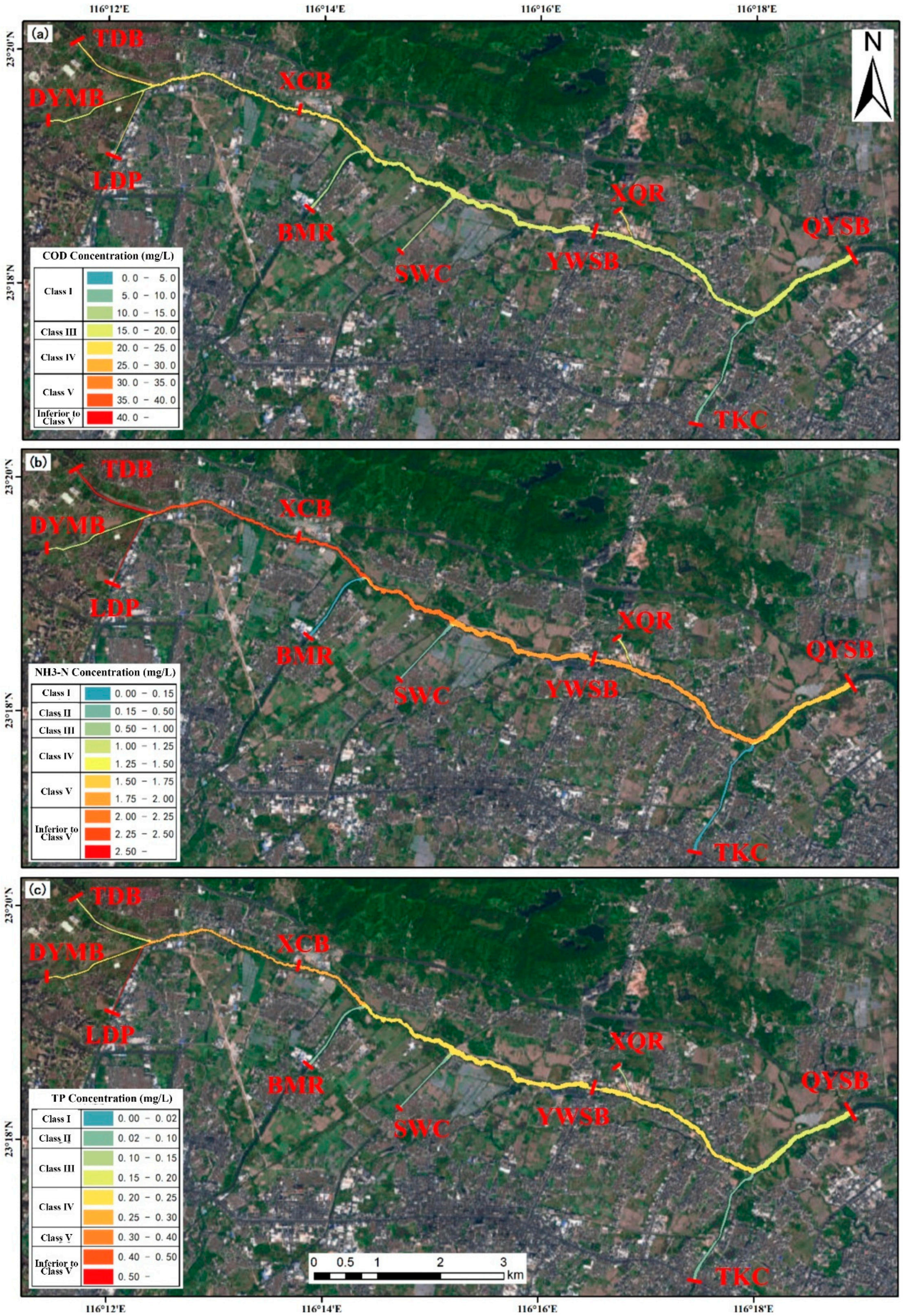

Fig. S23. Spatial distribution of pollutant loads under Scenario 1 (construction of a centralized textile-dyeing wastewater treatment center in the LSXR): (a) COD; (b)

NH3-N; (c) TP

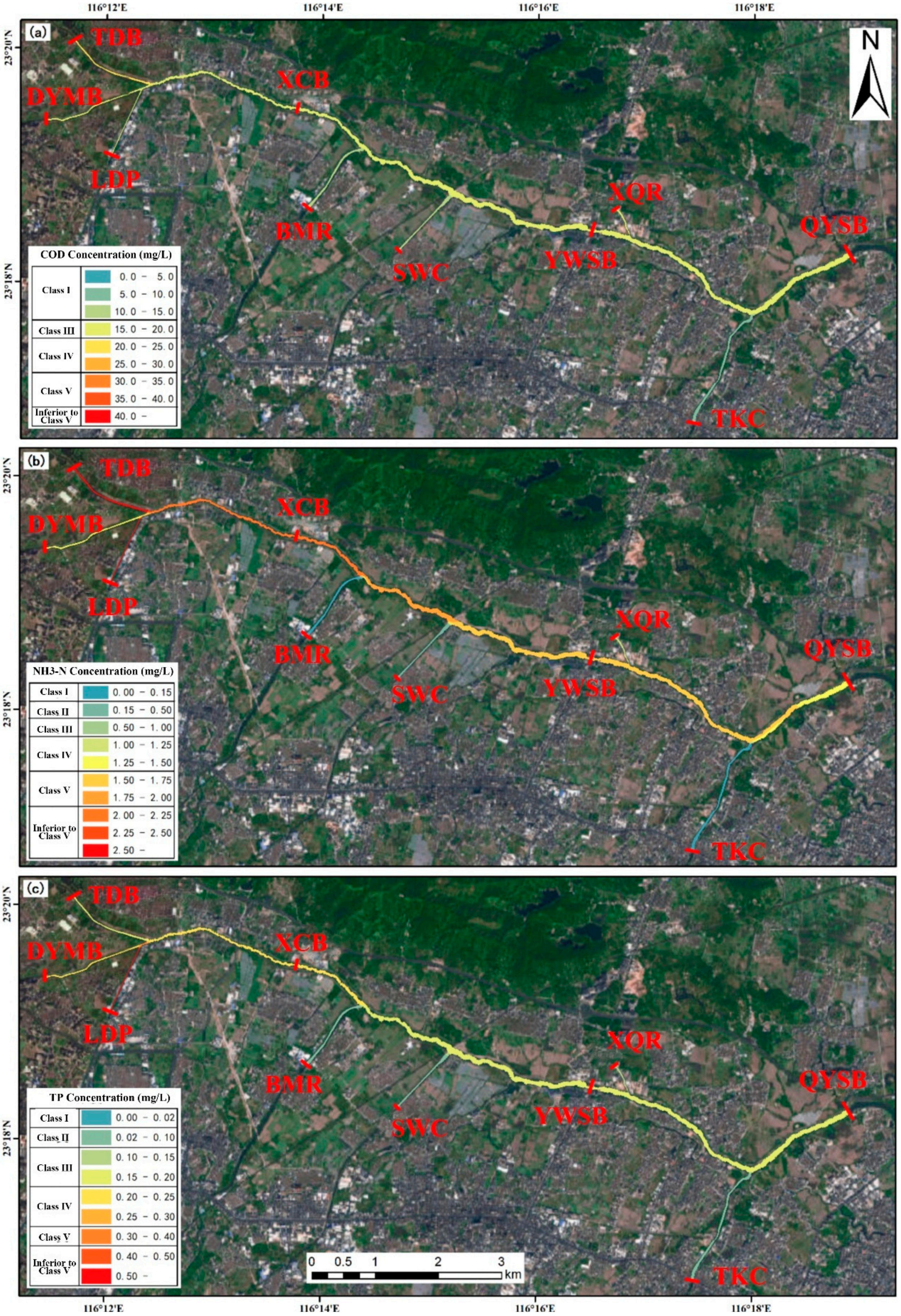

Fig. S24. Spatial distribution of pollutant loads under Scenario 2 (sewer network upgrading in the urban reach of Puning City, Lianjiang River Basin): (a) COD; (b) NH3-N; (c) TP

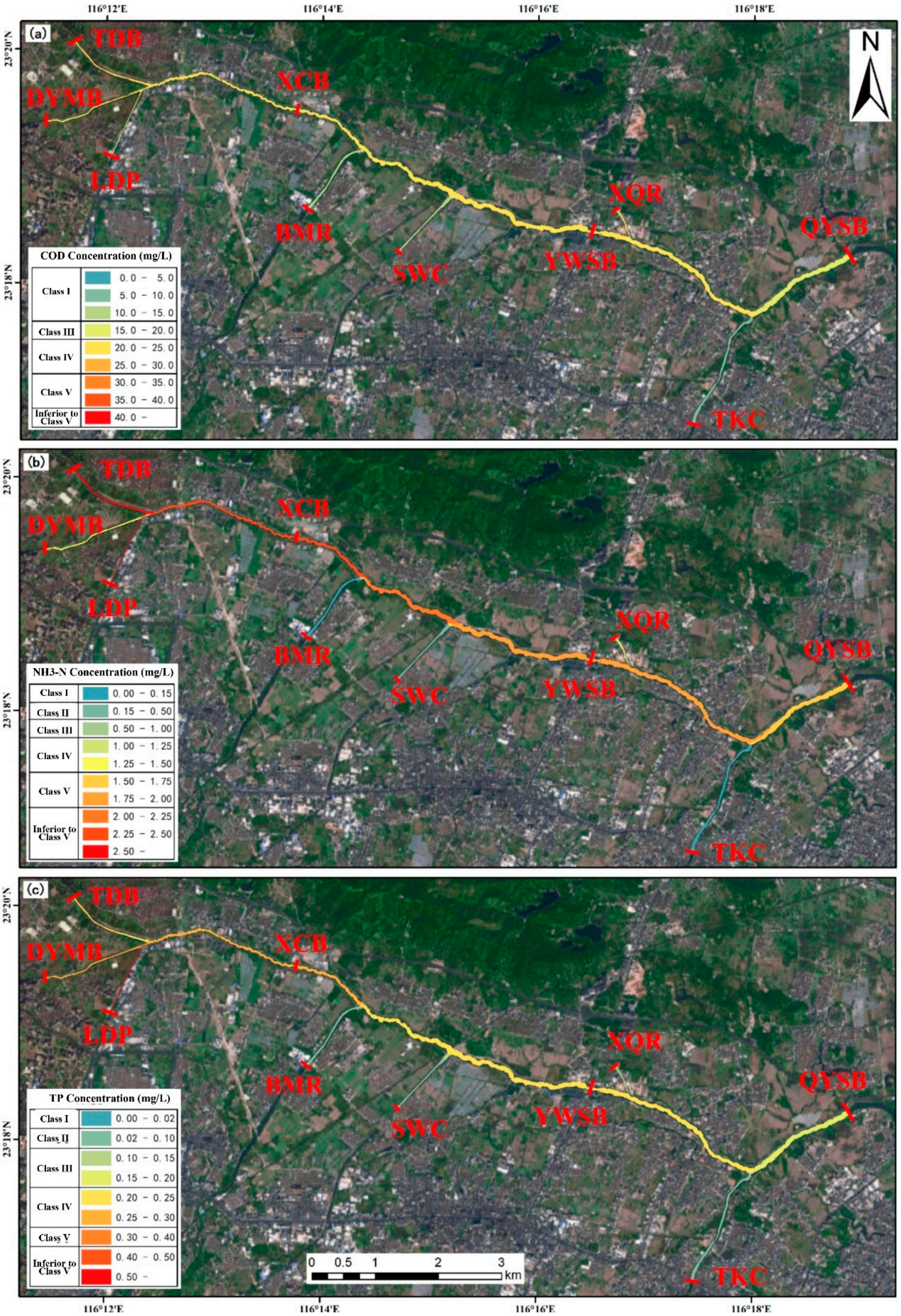

Fig. S25. Spatial distribution of pollutant loads under Scenario 3-1 (construction of a new wastewater treatment plant in the urban reach of Puning City, Lianjiang River Basin): (a) COD; (b) NH_3-N; (c) TP

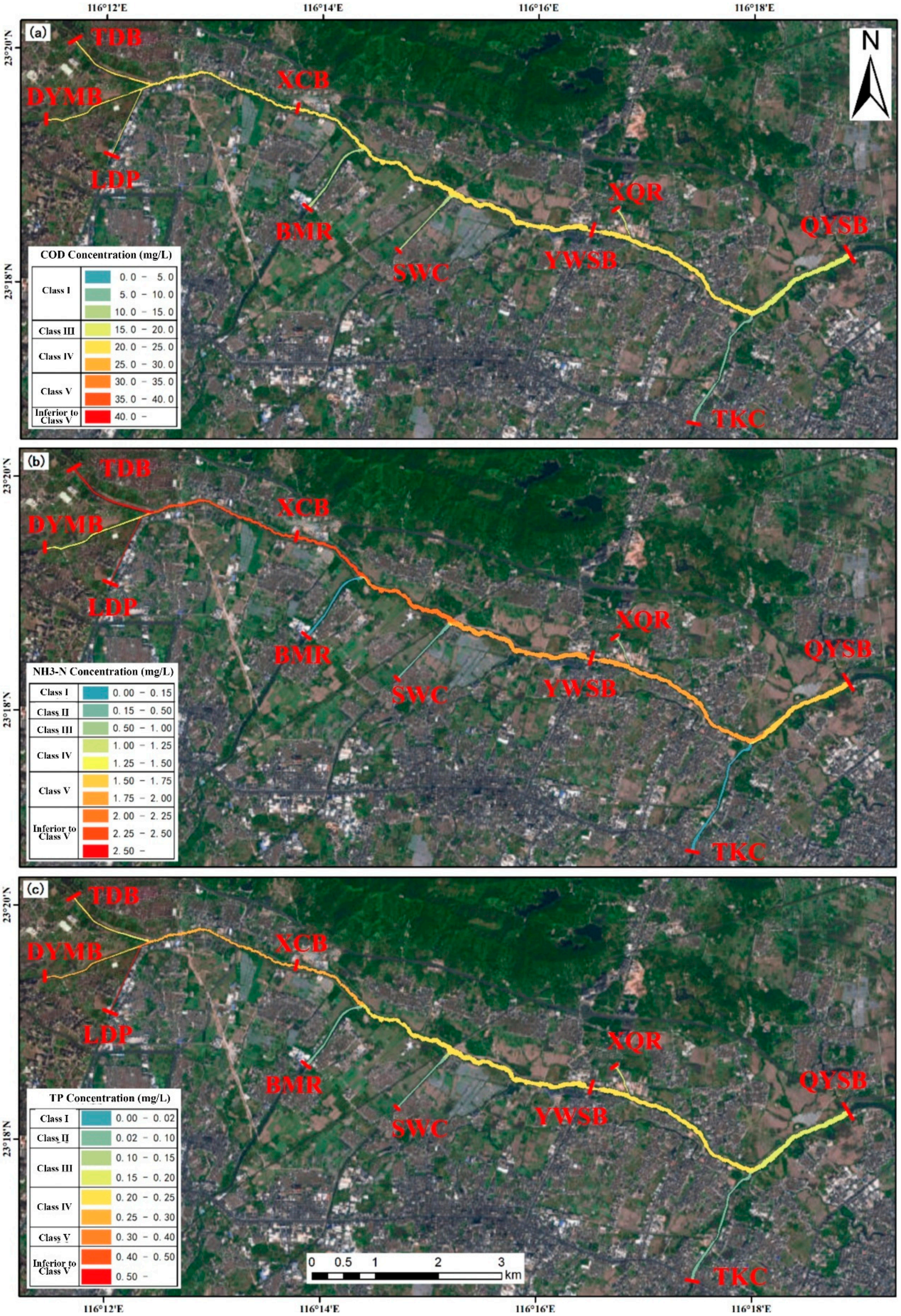

Fig. S26. Spatial distribution of pollutant loads under Scenario 3-2 (construction of a new wastewater treatment plant in the urban reach of Puning City, Lianjiang River Basin): (a) COD; (b) NH_3–N; (c) TP

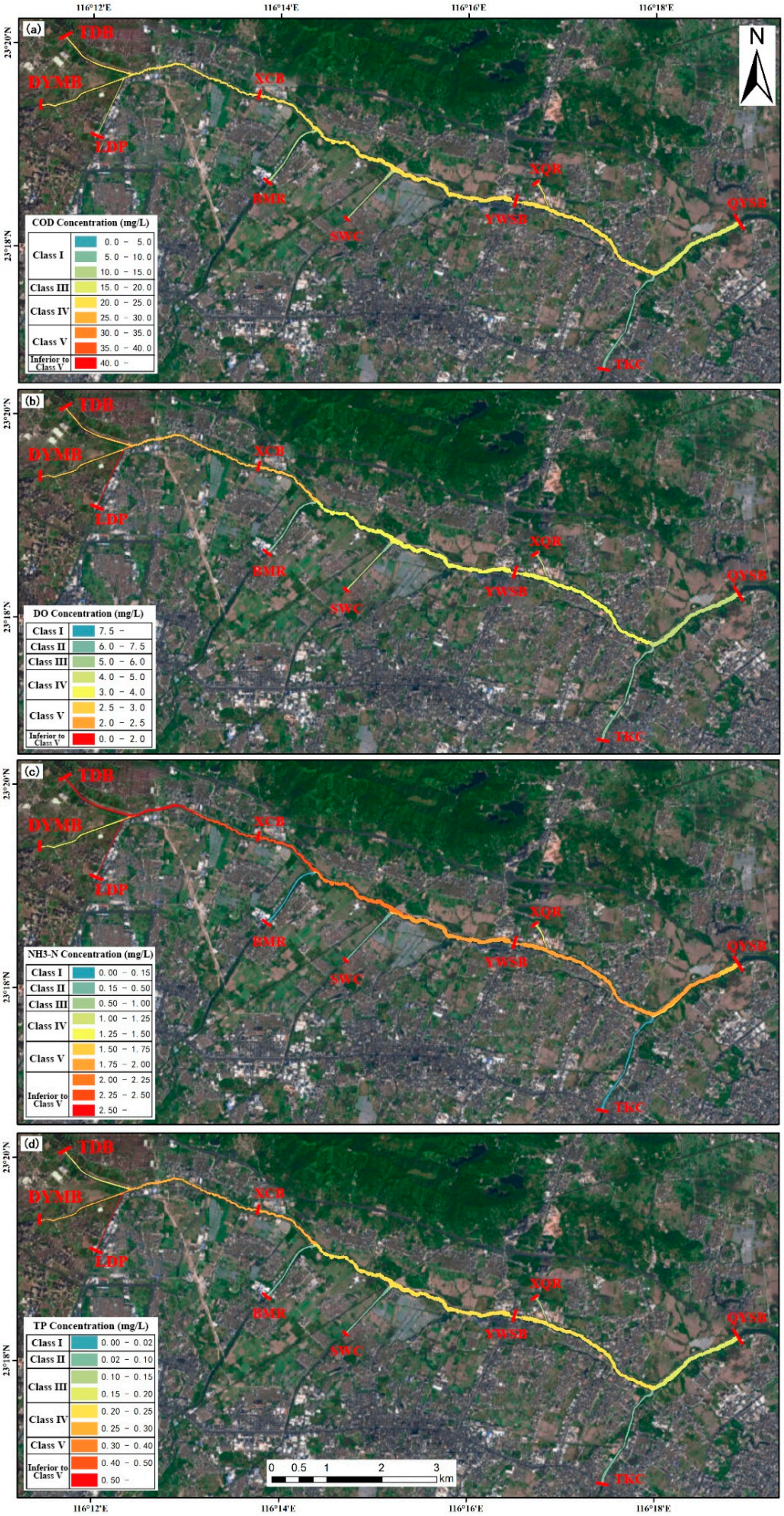

Fig.S27. Spatial distribution of pollutant loads under Scenario 4 (ecological water replenishment in the LSXR and the LSZR): (a) COD; (b) DO; (c) NH_3–N; (d) TP

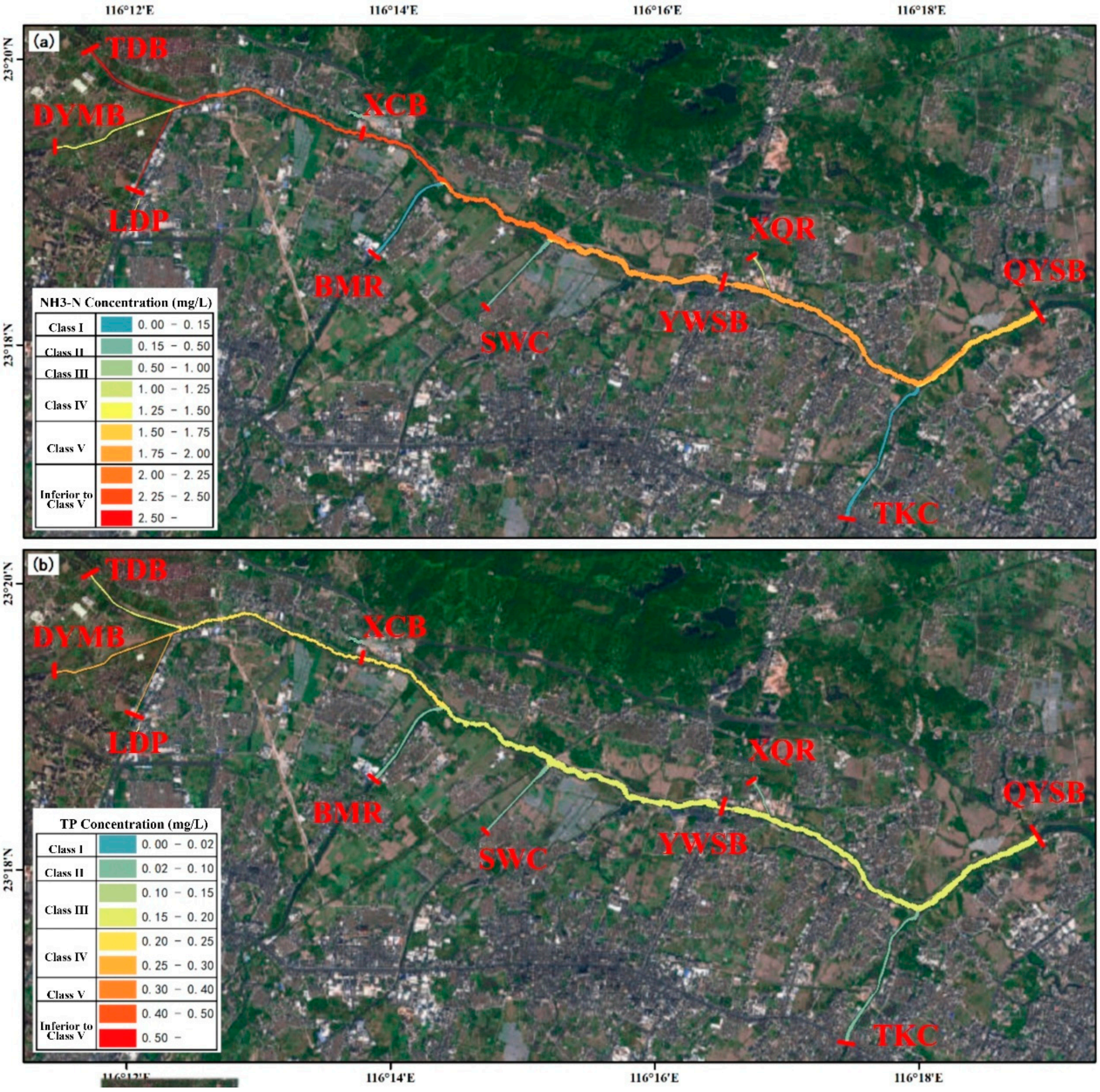

Fig.S28. Spatial distribution of pollutant loads under Scenario 5 (river dredging in the urban reach of Puning City, Lianjiang River Basin): (a) NH_3-N; (b) TP

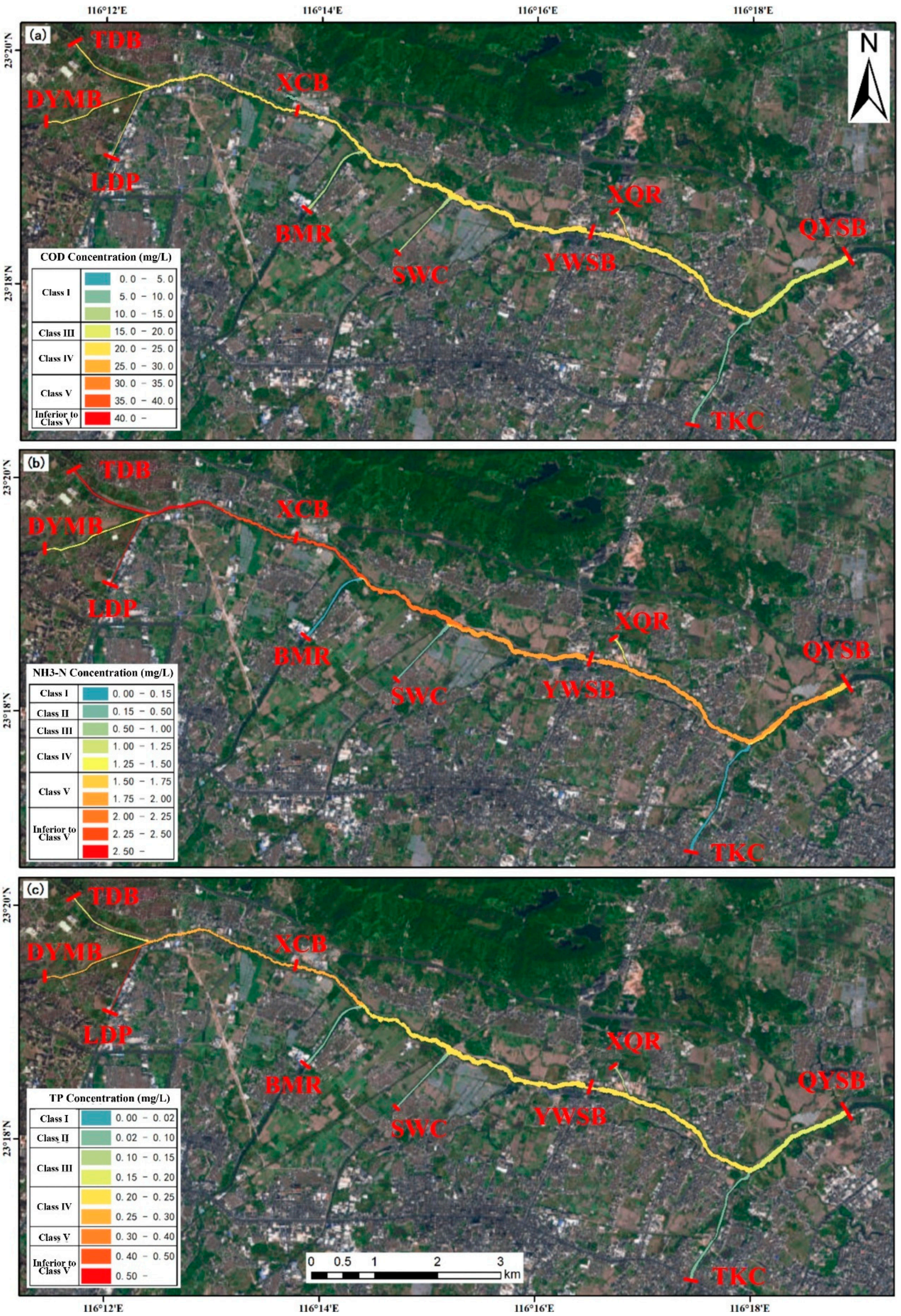

Fig. S29. Spatial distribution of pollutant loads under Scenario 6 (construction of ecological buffer strips along the BMR and the SWC): (a) COD; (b) NH_3–N; (c) TP

Text S6 Comparative assessment of management scenarios

Table S9. Pollutant reduction efficiencies of 11 interception-based management scenarios (unit: %)

Scenarios	Pollutants	XCB	YWSB	QYSB
Scenario 1	COD	57.81	51.01	47.21
	NH_3-N	65.69	64.00	63.16
	TP	41.39	39.72	37.28
Scenario 2	COD	29.40	26.30	24.01
	NH_3-N	18.21	16.62	17.51
	TP	28.97	28.02	26.09
Scenario 3	COD	12.79	11.85	10.44
	NH_3-N	16.10	14.52	15.48
	TP	29.63	28.64	26.69
Scenario 4	COD	0.00	8.81	8.46
	NH_3-N	0.00	0.98	0.96
	TP	0.00	2.92	2.86
Scenario 5	COD	0.00	3.15	3.08
	NH_3-N	0.00	0.86	0.75
	TP	0.00	2.02	2.02
Scenario 6	COD	0.00	0.00	2.17
	NH_3-N	0.00	0.00	1.44
	TP	0.00	0.00	1.29
Scenario 7	COD	0.00	0.00	4.62
	NH_3-N	0.00	0.00	0.69
	TP	0.00	0.00	3.77
Scenario 8	COD	87.21	74.19	71.22
	NH_3-N	83.90	81.85	80.67
	TP	70.37	64.79	63.37
Scenario 9	COD	70.59	60.06	57.65
	NH_3-N	81.79	79.80	78.64
	TP	71.03	65.40	63.96
Scenario 10	COD	42.19	35.90	34.46
	NH_3-N	34.31	33.47	32.99
	TP	58.61	53.96	52.78
Scenario 11	COD	100.00	85.07	81.67
	NH_3-N	100.00	97.56	96.15
	TP	100.00	92.08	90.05

Table S10. Pollutant reduction efficiencies of four engineering implementation schemes (unit: %)

Scenarios	Pollutants	XCB	YWSB	QYSB
Scenario 1	COD	26.68	22.14	21.79
	NH_3-N	32.93	33.15	31.66

	TP	30.23	27.30	27.22
Scenario 2	COD	22.01	18.13	17.97
	NH_3-N	17.35	18.18	16.68
	TP	11.77	10.17	10.60
Scenario 3	COD	4.68	3.28	3.83
	NH_3-N	15.58	16.49	14.98
	TP	18.46	16.38	16.62
Scenario 4	COD	49.37	41.58	40.32
	NH_3-N	50.82	50.35	48.86
	TP	42.39	38.57	38.17

Table S11. Pollutant reduction efficiencies of four engineering implementation schemes (unit: %)

Scenarios	Pollutants	XCB	YWSB	QYSB
Scenario 1	COD	7.61	6.47	6.22
	NH_3-N	4.69	4.58	4.51
	TP	2.57	2.37	2.31
Scenario 2	COD	15.00	14.56	15.00
	NH_3-N	15.00	14.90	15.00
	TP	15.00	14.56	15.00
Scenario 3-1	COD	1.37	1.16	1.12
	NH_3-N	2.47	2.41	2.37
	TP	-0.29	-0.26	-0.26
Scenario 3-2	COD	2.11	1.79	1.92
	NH_3-N	3.29	3.21	3.55
	TP	-0.05	-0.05	-0.34
Scenario 4	COD	11.66	8.11	6.38
	DO	27.47	16.86	15.79
	NH_3-N	24.77	20.13	17.46
	TP	3.53	1.18	-3.44
Scenario 5	COD	0.00	0.00	0.00
	NH_3-N	0.17	0.46	0.95
	TP	17.48	16.74	17.49
Scenario 6	COD	0.00	1.80	1.73
	NH_3-N	0.00	0.52	0.51
	TP	0.00	1.39	1.36
